# Novel Therapeutic Insights in Dedifferentiated Liposarcoma: A Role for FGFR and MDM2 Dual Targeting

**DOI:** 10.3390/cancers12103058

**Published:** 2020-10-20

**Authors:** Bérengère Dadone-Montaudié, Audrey Laroche-Clary, Aline Mongis, Emmanuel Chamorey, Ilaria Di Mauro, Vanessa Chaire, Pascal Finetti, Renaud Schiappa, François Le Loarer, Isabelle Birtwisle-Peyrottes, Jean-François Michiels, François Bertucci, Florence Pedeutour, Antoine Italiano, Laurence Bianchini

**Affiliations:** 1 Institute for Research on Cancer and Aging, Nice (IRCAN), Institut National de la Santé et de la Recherche Médicale (INSERM) U1081, Centre National de la Recherche Scientifique (CNRS) UMR7284, Université Côte d’Azur, 06107 Nice, France; dadone.b@chu-nice.fr (B.D.-M.); aline.mongis@univ-cotedazur.fr (A.M.); dimauro.i@chu-nice.fr (I.D.M); florence.pedeutour@univ-cotedazur.fr (F.P.); 2 Laboratoire de Génétique des Tumeurs Solides, Centre Hospitalier Universitaire de Nice, 06107 Nice, France; 3 Laboratoire Central d’Anatomie Pathologique, Centre Hospitalier Universitaire de Nice, 06001 Nice, France; michiels.jf@chu-nice.fr; 4 Institut National de la Santé et de la Recherche médicale (INSERM) U1218, Institut Bergonié, 33000 Bordeaux, France; a.laroche-clary@bordeaux.unicancer.fr (A.L.-C.); v.chaire@bordeaux.unicancer.fr (V.C.); f.le-loarer@bordeaux.unicancer.fr (F.L.L.); a.italiano@bordeaux.unicancer.fr (A.I.); 5 Unité de Biostatistiques, Centre Antoine Lacassagne, 06189 Nice, France; emmanuel.chamorey@nice.unicancer.fr (E.C.); renaud.schiappa@nice.unicancer.fr (R.S.); 6 Laboratoire d’Oncologie Prédictive, centre de Recherche en Cancérologie de Marseille, INSERM U1068, CNRS UMR7258, Institut Paoli-Calmettes, Université d’Aix-Marseille, 13009 Marseille, France; finettip@ipc.unicancer.fr (P.F.); bertuccif@ipc.unicancer.fr (F.B.); 7 Département de Biopathologie, Institut Bergonié, 33000 Bordeaux, France; 8 Laboratoire d’Anatomie Pathologique, Centre Antoine Lacassagne, 06189 Nice, France; isabelle.peyrottes@nice.unicancer.fr; 9 Département d’Oncologie Médicale, Institut Bergonié, 33000 Bordeaux, France; 10 Faculté de Médecine, Université de Bordeaux, 33400 Bordeaux, France

**Keywords:** liposarcoma, FGFR, MDM2, JNJ42756493, RG7388

## Abstract

**Simple Summary:**

Well-differentiated/dedifferentiated liposarcomas (WDLPS/DDLPS) are the most frequent soft tissue sarcomas. Despite the hopes raised by some targeted therapies, effective well-tolerated treatments for DDLPS are still lacking. Small-molecule FGFR inhibitors are currently evaluated in advanced clinical trials including the potent FDA-approved pan-FGFR inhibitor erdafitinib. We provide the first analysis of FGFR1-4 expression and their prognostic value in a series of 694 WDLPS/DDLPS samples. We identified FGFR1 and FGFR4 as prognostic biomarkers. We demonstrated erdafitinib efficacy and showed that erdafitinib combination with the MDM2 antagonist idasanutlin was highly synergistic in vitro and in vivo. The clinical relevance of our findings was supported by our data on a patient with DDLPS refractory to multiple lines of treatment whose tumor was stabilized for 12 weeks on erdafitinib. These data provide a rationale to use FGFR expression as a biomarker to select patients for clinical trials investigating FGFR inhibitors and to test combined erdafitinib and idasanutlin.

**Abstract:**

We aimed to evaluate the therapeutic potential of the pan-FGFR inhibitor erdafitinib to treat dedifferentiated liposarcoma (DDLPS). FGFR expression and their prognostic value were assessed in a series of 694 samples of well-differentiated/dedifferentiated liposarcoma (WDLPS/DDLPS). The effect of erdafitinib—alone or in combination with other antagonists—on tumorigenicity was evaluated in vitro and in vivo. We detected overexpression of FGFR1 and/or FGFR4 in a subset of WDLPS and DDLPS and demonstrated correlation of this expression with poor prognosis. Erdafitinib treatment reduced cell viability, inducing apoptosis and strong inhibition of the ERK1/2 pathway. Combining erdafitinib with the MDM2 antagonist RG7388 exerted a synergistic effect on viability, apoptosis, and clonogenicity in one WDLPS and two DDLPS cell lines. Efficacy of this combination was confirmed in vivo on a DDLPS xenograft. Importantly, we report the efficacy of erdafitinib in one patient with refractory DDLPS showing disease stabilization for 12 weeks. We provide evidence that the FGFR pathway has therapeutic potential for a subset of DDLPS and that an FGFR1/FGFR4 expression might constitute a powerful biomarker to select patients for FGFR inhibitor clinical trials. In addition, we show that combining erdafitinib with RG7388 is a promising strategy for patients with DDLPS that deserves further investigation in the clinical setting.

## 1. Introduction

Sarcomas are rare solid tumors of mesenchymal origin. They constitute a heterogeneous and complex group of approximately 70 pathological subtypes. Among them, liposarcomas (LPS), derived from a lipogenic origin, are divided into four subtypes, according to the World Health Organization (WHO) Classification: atypical lipomatous tumor (ALT)/well-differentiated LPS (WDLPS), dedifferentiated LPS (DDLPS), myxoid LPS, and pleomorphic LPS. ALT/WDLPS are only locally aggressive, but, in 10% of the cases, they recur and dedifferentiate and therefore may acquire the same metastatic potential and poor prognosis as de novo DDLPS [1,2]. Even though the “LPS” subtype has been identified as a favorable prognostic factor in advanced soft tissue sarcomas [3], and, although new cytotoxic agents have been developed such as trabectedin [4] and eribulin [5], the currently available chemotherapy regimens remain poorly efficient in advanced WDLPS/DDLPS [6].

With respect to targeted therapies, the pazopanib anti-angiogenic drug represents the only targeted therapy currently approved in soft tissue sarcomas but is not indicated in LPS because of unproven efficiency in the PALETTE trial [7]. Both WDLPS and DDLPS are characterized by the amplification of a discontinuous region on the long arm of the chromosome 12 [1,2]. This 12q13–15 amplicon encompasses several oncogenes including *MDM2*, *CDK4*, and *HMGA2* [1,2,8,9,10], as well as *CPM* (*Carboxypeptidase M*), a putative oncogene involved in the EGFR pathway [11] thus retained as potential therapeutic targets. The discovery of the *MDM2* amplification in 100% of WDLPS and DDLPS cases [9] led to the development of targeted therapies able to restore the pro-apoptotic effect of p53, by inhibiting the MDM2-p53 binding [12,13] including the nutlins. *CDK4* amplification led to the testing of CDK4 inhibitors, palbociclib, and abemaciclib, in locally advanced and metastatic DDLPS [14,15]. Tumor responses were observed in these early-phase trials and the development of these drugs is ongoing. 

Another gene almost always amplified in WDLPS and DDLPS is *Fibroblast Growth Factor Receptor Substrate 2* (*FRS2*), located close to *MDM2* (at approximately 600kb) [9,16,17]. *FRS2* plays a key role in the Fibroblast Growth Factor (FGF)/Fibroblast Growth Factor Receptor (FGFR) signaling pathway, acting as an adaptor protein to activate the RAS/RAF/MAPK and the PI3K/AKT signaling cascades, leading to cell proliferation, migration, and survival [18]. Aberrant activation of this tyrosine kinase receptor, consecutively to a FGFR alteration (i.e., amplification, mutation, or fusion) or an overexpression of FGF ligands, has been demonstrated in several types of cancers, such as bladder, breast, or lung carcinoma [19]. Despite the observation of the recurrent *FRS2* amplification, very few data are available about the FGFR expression [20,21,22] and the FGFR pathway deregulation in WDLPS and DDLPS [17,23,24]. Frequent constitutive FGFR activation made FGFRs attractive targets for anti-cancer therapies. Several FGFR inhibitors are currently under clinical evaluation. Notably, erdafitinib (JNJ42756493), a potent and very selective pan-FGFR tyrosine kinase inhibitor (TKI) [25], has been approved for patients with locally advanced or metastatic urothelial carcinoma, with susceptible *FGFR3* or *FGFR2* genetic alterations, which has progressed during or following platinum-containing chemotherapy [26]. Regarding soft tissue sarcomas, phase II clinical trials investigating multi-kinase inhibitors targeting FGFRs among other receptor tyrosine kinases such as sunitinib, sorafenib, regorafenib, and anlotinib have shown some efficacy depending on the histological subtype [27,28,29,30,31]. Concerning the specific FGFR inhibitors, the following clinical trials are underway: a phase 2 basket trial on different types of cancers including some sarcomas testing Erdafitinib (NCT03210714, https://clinicaltrials.gov/ct2/show/NCT03210714) and a phase 3 trial, MULTISARC, specific for advanced sarcomas testing TAS-120, a selective pan-FGFR TKI (NCT03784014, https://clinicaltrials.gov/ct2/show/NCT03784014).

The main objective of our study was to determine the effect of the inhibition of the FGFR pathway in the treatment of DDLPS. We first analyzed the FGFRs expression and their prognostic value in a series of 694 clinical samples of WDLPS and DDLPS. We were also able to evaluate the efficacy of erdafitinib treatment in a patient of this cohort with refractory DDLPS. Monotherapies often lack sustained efficiency and lead to the emergence of secondary drug resistance; we therefore explored the potential synergy of two different combinations: first between erdafitinib and the PI3K/mTOR antagonist BEZ235; second between erdafitinib and idasanutlin (RG7388), a potent and selective antagonist of the MDM2-p53 binding [32].

## 2. Results

### 2.1. Expression of FGFRs in WDLPS/DDLPS Patients and Prognostic Impact

**Figure 1:**
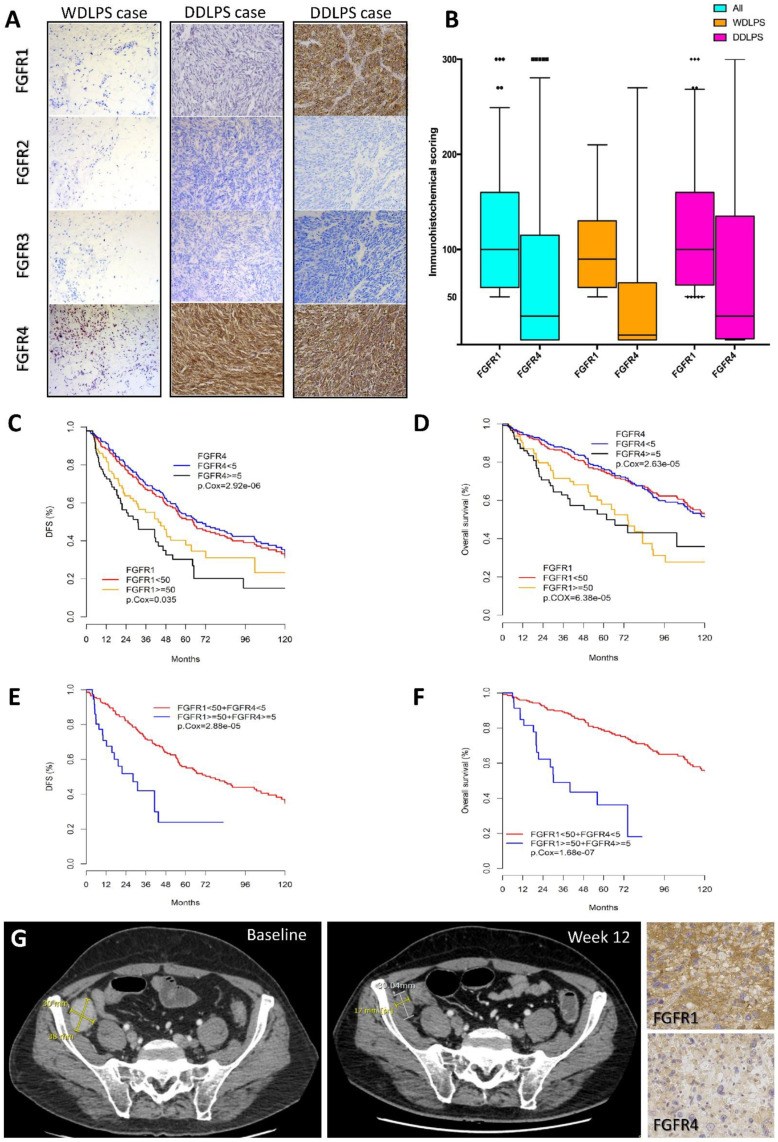
FGFR1 and FGFR4 expression in WDLPS/DDLPS clinical tumor samples and prognostic values. (**A**,**B**) Immunohistochemical analysis in our series of 418 tumors. (**A**) Representative illustrations of WDLPS cases positive for FGFR4 (left-hand side); DDLPS cases positive for FGFR4 (middle column) and DDLPS cases positive for both FGFR1 and FGFR4 expression (right-hand side) (localization: membrane and cytoplasm, magnification ×100). (**B**) Box plot showing the distribution of IHC scores for the overexpressing cases* for FGFR1 and FGFR4 in the WDLPS and DDLPS cases of the IHC cohort. (**C–F**) Prognostic value of FGFR1 and FGFR4 expression in our cohort of 358 WDLPS/DDLPS patients: Kaplan-Meier survival analyses of FGFR1 and/or FGFR4 expression shows correlation between FGFR1 and/or FGFR4 expression and disease-free survival (DFS, C and E) and overall survival (OS, D and F). (**G**) Effects of treatment with the erdafitinib pan-FGFR inhibitor (JNJ42756493) in a patient with refractory DDLPS. CT scan images of a 57-year-old male patient with a refractory DDLPS before and after erdafitinib treatment. The patient was resistant to doxorubicin, regorafenib, high-dose ifosfamide, trabectedin and presented a stable disease during 12 weeks (according to the RECIST criteria) under erdafitinib treatment. This tumor co-expressed FGFR1 (score 120) and FGFR4 (score 60).

First, we assessed FGFR1–4 protein expression in a series of 418 cases from 358 patients including a majority of DDLPS cases (252 DDLPS versus 106 WDLPS cases). IHC analysis was performed on the primitive tumor in the vast majority of cases (79%), on recurrence in 19%, and on metastasis in 2% of the cases. For 47 patients, at least two samples were analyzed. Patient clinicopathological characteristics are summarized in Table S1. The median follow-up was 78.1 months (95% CI (65.3–94.6)). Immunohistochemistry (IHC) analysis showed that none of the tumors expressed FGFR2 or FGFR3 (Figure 1A). Overexpression of FGFR1 was detected in 27% of all the LPS cases, overexpression of FGFR4 in 27% of the cases, and co-overexpression of FGFR1 and FGFR4 in 11% of the cases (Table 1). Representative examples of positive staining for FGFR1 and FGFR4 in WDLPS and DDLPS cases are shown in Figure 1A. For both FGFR1 and FGFR4, overexpression was markedly more frequent in DDLPS than in WDLPS (34% of DDLPS vs. 11% of WDLPS for FGFR1; 32% of DDLPS vs. 14% of WDLPS for FGFR4). In these overexpressing cases, the median score was 100 for FGFR1 and 30 for FGFR4 (Figure 1B). Out of the 47 patients for which more than one sample was analyzed, IHC was performed on the primitive tumor and on its recurrence(s) for 35 patients. For 23 cases, the same level of FGFR1 and/or FGFR4 expression was detected between the primitive tumor and the recurrences.

Interestingly, we observed that, for five cases, FGFR expression either appeared or was more intense in the recurrent tumor than in the primitive one; three of these patients were deceased and the remaining two patients had progressive disease.

We then assessed the prognostic value of FGFR1 and/or FGFR4 overexpression in this cohort of 358 patients. FGFR1 and/or FGFR4 overexpression was associated with shorter disease-free survival (DFS) and overall survival (OS). Regarding DFS, univariate analysis (Table S2) showed a higher risk of recurrence for patients with FGFR1 overexpression (Hazard ratio, HR = 1.4, 95%CI [1.0–2.0], *p* = 0.03; Figure 1C), FGFR4 overexpression (HR = 2.2, 95% CI [1.6–3.1], *p* < 0.0001; Figure 1C), and FGFR1 + FGFR4 co-overexpression (HR = 2.9, 95% CI [1.8–4.9], *p* < 0.0001; Figure 1E). Pathological subtype and FNCLCC grading were also associated with prognosis. Multivariate analysis confirmed that FGFR4 overexpression (HR = 1.9, 95% CI [1.4–2.7], *p* = 0.00015) and FGFR1 + FGFR4 co-overexpression (HR = 2.1, 95% CI [1.3–3.4], *p* = 0.0025) were independent adverse prognostic factors (Table S2). Similar results were observed for OS. Cox univariate analysis (Table S2) showed a higher risk of death for patients with FGFR1 overexpression with HR equal to 2.1 (95% CI [1.5–3.0], *p* < 0.0001; Figure 1D), and for patients with FGFR4 overexpression (HR = 2.3, 95% CI [1.6–3.4], *p* < 0.0001; Figure 1D). The death risk was even higher when FGFR1 + FGFR4 were co-overexpressed (HR = 4.7, 95% CI [2.6–8.4], *p* < 0.0001; Figure 1F). Non-complete resection (i.e., R1 or R2 resection), pathological subtype and location were also associated with poor prognosis. Cox multivariate analysis revealed that FGFR1 overexpression (HR = 1.9, 95% CI [1.2–3.0], *p* = 0.008), FGFR4 overexpression (HR = 2.0, 95% CI [1.2–3.1], *p* = 0.006) and FGFR1 + FGFR4 co-overexpression (HR = 4.0, 95% CI [2.1–7.8], *p* < 0.0001) remained independent adverse prognostic factors for OS (Table S2).

In order to validate these results in an independent series of patients, we examined *FGFR1* and *FGFR4* mRNA expression in our pooled data set of 58 WDLPS and 218 DDLPS samples and 11 normal fat samples [33]. Their clinicopathological characteristics are summarized in Table S3. The proportions of each LPS subtype were close to those of the “IHC” series with a majority of DDLPS (79% vs. 21%). Expression was heterogeneous across samples. Similar to protein expression, mRNA expression of *FGFR1* was higher than that of *FGFR4* in both WDLPS and DDLPS (Figure S1A). As observed at the protein level, a subset of LPS samples overexpressed *FGFR1* and *FGFR4*: 62 out of 276 LPS (22%) showed *FGFR1* overexpression, 51 out of 276 (18%) showed *FGFR4* overexpression, and 25 out of 276 (9%) showed *FGFR1 + FGFR4* co-overexpression. In univariate analysis for DFS, *FGFR1* and/or *FGFR4* overexpression were also associated with shorter DFS (Figure S1B–D). The HR for DFS event in patients with overexpression when compared to patients without overexpression was 2.4 (95%CI [1.4–3.9]) for *FGFR1* (*p* = 0.00069), 1.9 (95%CI [1.1–3.3]) for *FGFR4* (*p* = 0.029), and 2.6 (95%CI [1.4–4.9]) for the *FGFR1 + FGFR4* co-overexpression (*p* = 0.0028), thus confirming the results observed at the protein level. In contrast, we found no correlation between expression of *FGFR2*, *FGFR3,* or *FRS2* and prognosis (Figure S1E–G).

### 2.2. FGFR Targeting in a Patient with Refractory DDLPS

In order to further investigate the clinical relevance of our findings, we analyzed the case of one patient from our IHC cohort with advanced DDLPS treated with the pan-FGFR inhibitor erdafitinib. This 57-year-old patient with metastatic DDLPS refractory to four prior lines of chemotherapy and targeted therapy (doxorubicin, regorafenib, high-dose ifosfamide, and trabectedin) was referred to the early phase unit of Institut Bergonié. He was enrolled in a phase I study assessing the safety and efficacy of erdafitinib. After 6-week treatment, the CT-scan showed a 20% decrease of the target lesion (RECIST 1.1 stabilization) which persisted after 12 weeks (Figure 1G). At week 18, the patient presented disease progression and was withdrawn from the study. Importantly, in support of our hypothesis, analysis of FGFR1 and FGFR4 protein expression of the patient tumor showed co-overexpression of FGFR1 (IHC score 120) and FGFR4 (IHC score 60) (Figure 1G). CGH/SNP-array analysis of the tumor showed that co-overexpression of FGFR1 and FGFR4 did not result from *FGFR* gene amplification.

### 2.3. Involvement of the FGF/FGFR Signaling Pathway in LPS Tumorigenesis

As illustrated by the case of our patient treated with erdafitinib, tumors almost systematically escape monotherapies even when they are targeted, which underscores the need to identify appropriate combinations. We used a panel of in-house well-characterized and validated WDLPS (93T449) and DDLPS (IB111 and IB115) cell lines to investigate the therapeutic potential of erdafitinib alone and in combination with other antagonists.

We first assessed FGFR1–4 and FRS2 expression both at the mRNA and protein levels using respectively qRT-PCR and immunoblotting. 

**Figure 2:**
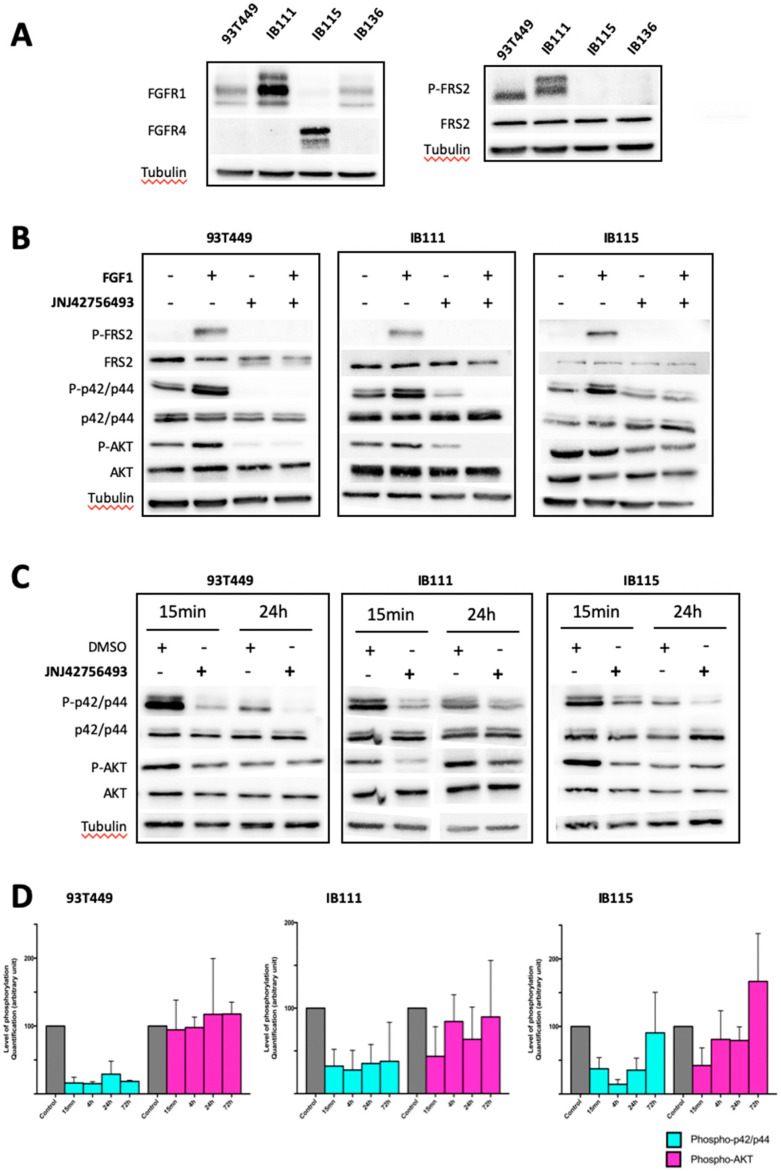
Analysis of the FGFR/FRS2 signaling pathway after FGFR stimulation and inhibition in WDLPS and DDLPS cell lines. Western blot analysis showing (**A**) The level of expression for the indicated proteins in one WDLPS (93T449) and two DDLPS (IB111 and IB115) cell lines was compared to the level of expression for the same proteins in a soft tissue leiomyosarcoma cell line (IB136) used as a negative control (non-adipose soft tissue sarcoma). (**B**) The level of phosphorylated and total protein for the indicated proteins in 93T449, IB111 and IB115 cells treated for 30 min with JNJ42756493 at the IC50 concentrations: 0.36 µM (93T449), 1 µM (IB111), and 2 µM (IB115), with or without FGF1 stimulation (10 ng/mL for 15 min) as indicated. (**C**) The level of phosphorylated and total protein for the indicated proteins in 93T449, IB111 and IB115 cells treated for 15 min and 24 h with JNJ42756493 at the IC50 concentrations: 0.36 µM (93T449), 1 µM (IB111), and 2 µM (IB115). In all Western blot experiments, β-tubulin was used as a loading control. (**D**) Quantification of the Western blot analyses shown in (**C**) performed on cells treated with JNJ42756493 for 15 min, 4 h, 24 h, and 72 h.

Expression of FGFR2 and FGFR3 was very low at the mRNA level (Figure S2A) and not detectable at the protein level in WDLPS and DDLPS cells. In contrast, *FGFR1* mRNA was the most abundantly expressed FGFR in WDLPS and DDLPS cells at comparable levels to those detected in normal fat cells (Figure S2A). *FGFR4* expression was only detected in IB115 cells (Figure S2A). We found a consistent correlation between protein and mRNA expression for FGFR1 and FGFR4 (Figure 2A) demonstrating that WDLPS and DDLPS cells exhibit the same pattern of FGFR expression as primary tumors, namely predominant expression of FGFR1 and/or FGFR4. 

Consistent with previous reports [16,17], we detected higher levels of *FRS2* mRNA in LPS cells than in normal adipose tissue and in the IB136 LMS cells (Figure S2A), especially in the WDLPS cell line (93T449). However, mRNA did not appear to translate efficiently in protein since the level of FRS2 protein in WDLPS/DDLPS cells was not higher than in the IB136 leiomyosarcoma (LMS) cells (Figure 2A). In contrast, phosphorylated FRS2 was detectable in LPS cells, but not in LMS cells suggesting constitutive activation of the FGFR pathway in LPS cells (Figure 2A). Phosphorylated FRS2 was not systematically detected in IB115 unstimulated cells probably because it was at the limit of the detection with the antibody we used, as previously reported in other DDLPS cell lines [23]. 

We then studied the effects of JNJ42756493 (erdafitinib) on cell proliferation. Using an MTT assay, we first demonstrated that the three LPS cell lines of our panel were sensitive to this inhibitor with IC_50_ values ranging from 0.36 to 1–2 µM (Table S4 and Figure S2B). We further investigated the effect of the FGFR inhibitor on the different components of the FGFR signaling pathway from FRS2 to the downstream kinases p42/p44 MAPK and AKT both in unstimulated cells and in FGF1-treated cells. FGF1 treatment greatly increased FRS2 phosphorylation; p42/p44 MAPK phosphorylation was also stimulated although to a lesser extent, whereas the effect on AKT phosphorylation was even less pronounced and not consistent. Pretreatment with JNJ42756493 completely abolished FGF1-induced FRS2 phosphorylation and significantly reduced p42/p44 MAPK and AKT phosphorylation (Figure 2B). We further examined the effects of JNJ42756493 on activation of p42/p44 MAPK and AKT by exposing the cells to JNJ42756493 from 15 min to 72 h. As shown in Figure 2C,D, JNJ42756493 treatment for 15 min to 24 h significantly decreased p42/p44 MAPK phosphorylation, whereas the effect on AKT phosphorylation was much less pronounced and only short-term. When cells were exposed to JNJ42756493 for 72 h, we observed a rebound for p42/p44 MAPK phosphorylation in IB115 cells suggesting that feedback mechanisms might reactivate this pathway. As we did not observe a consistent inhibition of AKT phosphorylation upon JNJ42756493 treatment and because the involvement of the PI3K/AKT pathway in WDLPS/DDLPS tumorigenesis has been reported [34], we tested a specific dual inhibitor of the PI3K/AKT/mTOR pathway BEZ235. As expected, BEZ235 strongly inhibited AKT phosphorylation in the WDLPS and DDLPS cell lines (Figure S3A,B). The effect of the JNJ42756493 and BEZ235 combination was then tested on viability, apoptosis, and cell cycle in these cell lines (Figure S3C–E). Drug synergy assays and the synergy analysis were performed as previously described [35] using the isobologram and combination index (CI) methods derived from the median-effect principle of Chou and Talalay [36]. Synergy between the two drugs was not observed for the DDLPS cell lines (CI values superior to 1), and this lack of synergy was confirmed when the combination of JNJ42756493 and BEZ235 was tested on apoptosis induction and cell cycle distribution (Figure S3D,E). This led us to investigate the potential synergy of another combination.

### 2.4. Dual Targeting of FGFR and MDM2 in WDLPS/DDLPS Cell Lines

**Figure 3:**
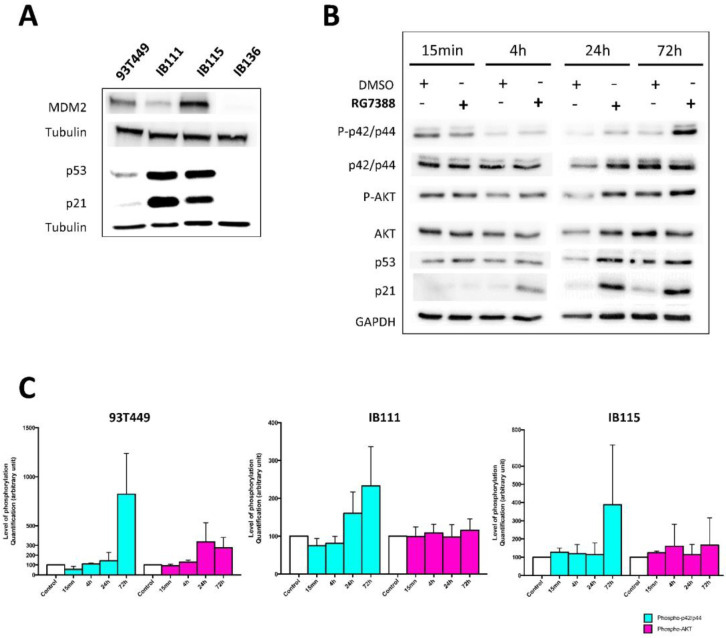
Effects of RG7388 as a single agent on activation of the downstream signaling pathways. (**A**) Western blot analysis showing the level of expression for the indicated proteins in 93T449, IB111, IB115 and IB136 cells. (**B**) Western blot analysis showing the level of phosphorylated and total protein for the indicated proteins in 93T449 cells treated for 15 min, 4 h, 24 h and 72 h with RG7388 at the IC50 concentration (0.04 µM). (**C**) Quantification of the western blot analyses shown in (**B**) and in the two other cell lines (IB111 and IB115) performed on cells treated with RG7388 for 15 min, 4 h, 24 h, and 72 h.

Consistent with *MDM2* amplification and overexpression being a hallmark of WDLPS/DDLPS tumors and cell lines, we detected MDM2 overexpression in WDLPS and DDLPS cells but not in the IB136 LMS cells (Figure 3A and Figure S2A). We also showed that, in comparison to IB136 cells, DDLPS cells and, to a lesser extent, WDLPS cells overexpress p53 and p21 (Figure 3A). In WDLPS and DDLPS tumors, MDM2 overexpression status, while retaining wild-type p53, allowed the development of targeted therapies able to restore the pro-apoptotic effect of p53, such as nutlins. We had reported that the nutlin analog RG7388 exerted anti-tumor effects in DDLPS cells [35]. Moreover, inhibition of ERK signaling using a MAPK kinase inhibitor was demonstrated to exert a synergistic effect with RG7388 on tumor growth in DDLPS [37] providing a rationale to investigate the potential of dual targeting of FGFR and MDM2 in WDLPS and DDLPS cells. 

We determined the sensitivity of the three LPS cell lines to RG7388 and found IC_50_ values in the nanomolar range from 40 to 200 nM (Table S4). RG7388 treatment resulted in an accumulation of the p53 protein and of its target p21 but also induced an upregulation of phosphorylated p42/p44 MAPK (Figure 3B,C) in WDLPS and DDLPS cells (as already observed in DDLPS cells [37]). 

**Figure 4:**
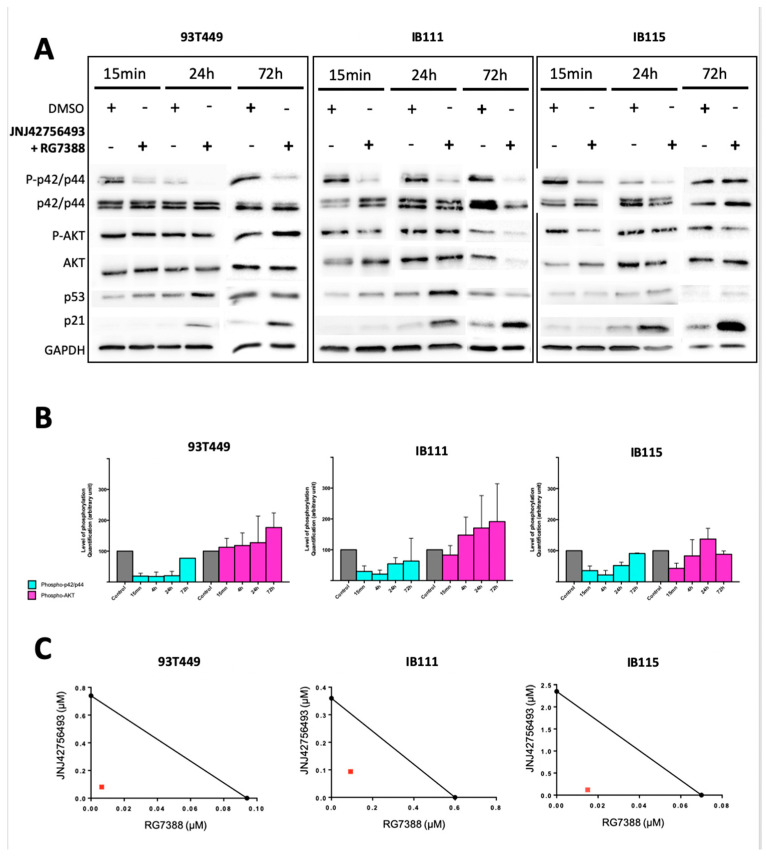
Effects of RG7388 in combination with JNJ42756493 on activation of the downstream signaling pathways. (**A**) Western blot analysis showing the level of phosphorylated and total protein for the indicated proteins in 93T449, IB111 and IB115 cells treated for 15 min, 24 h and 72 h with JNJ42756493 at 0.36 μM (93T449), 1 μM (IB111), and 2 μM (IB115) and RG7388 at 0.04 μM (93T449), 0.2 μM (IB111), and 0.05 μM (IB115). (**B**) Quantification of the western blot analyses shown in (**A**) performed on cells treated with JNJ42756493 + RG7388 for 15 min, 4 h, 24 h, and 72 h. (**C**) Isobologram representation for the 93T449, IB111 and IB115 cell lines. The combination index (CI) values for the JNJ42756493 + RG7388 combination were calculated and were the following: 0.18, 0.40 and 0.26 respectively, indicating synergy for both WDLPS and DDLPS cells.

We tested the potential synergism of the combination JNJ42756493 + RG7388 by determining the CI values [36]. As shown in the isobologram representation (Figure 4C), each cell line exhibited CI values inferior to 1: 0.18 (93T449), 0.40 (IB111) and 0.26 (IB115) indicating synergism for both WDLPS and DDLPS cells. 

We examined the effect of the JNJ42756493 + RG7388 combination on p42/p44 MAPK and AKT phosphorylation as well as on the expression of p53 and p21. The combined treatment induced an accumulation of the p53 protein and of p21 (Figure 4A). The effects we observed on p42/p44 MAPK and AKT were comparable to those we detected upon JNJ42756493 treatment alone (Figure 2C,D), mainly an inhibition of p42/p44 MAPK phosphorylation from 15 min to 24 h with a rebound at 72 h (Figure 4A,B).

**Figure 5:**
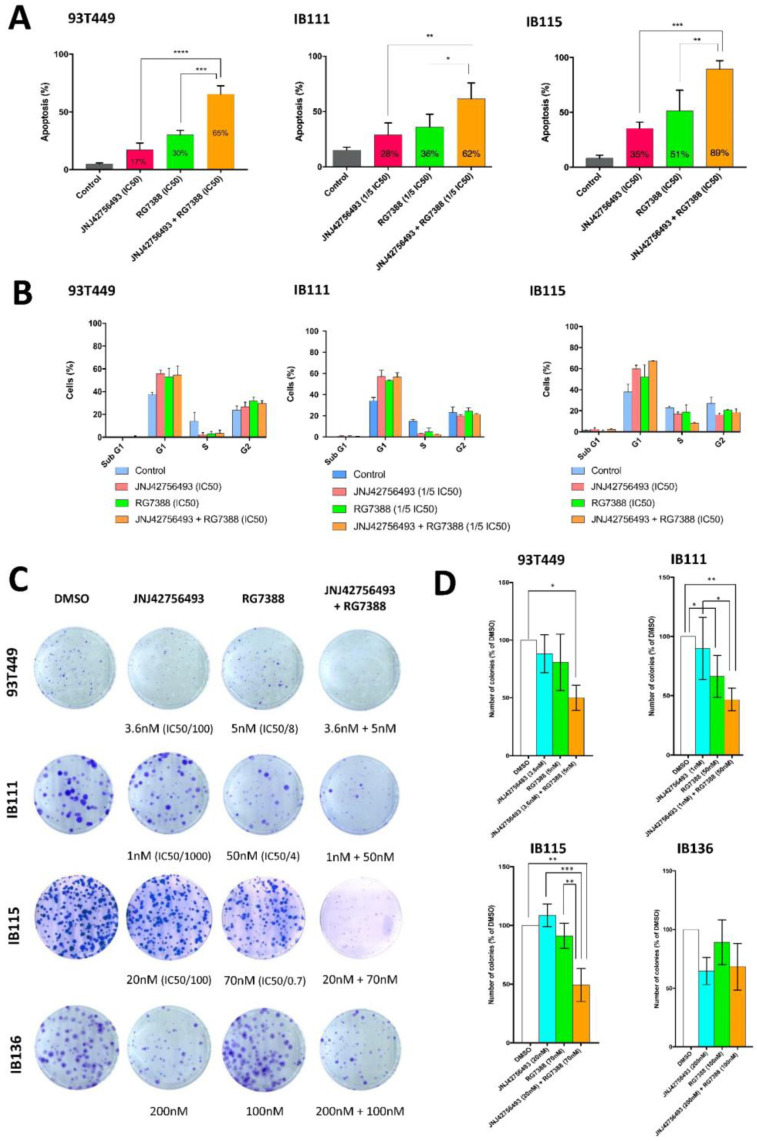
Effects of JNJ42756493 and RG7388, as single agents and in combination on in vitro tumorigenicity. (**A**) Effects on apoptosis induction. Quantification of apoptotic cells using annexin V-FITC and propidium iodide staining followed by flow cytometry after 72 h of treatment by JNJ42756493 or RG7388 and in combination (shown in Figure S4A). JNJ42756493 and RG7388 concentrations were determined according to IC_50_ values, respectively: 0.36 μM and 0.04 μM (93T449), 0.2 μM and 0.04 μM (for the IB111 cells, because of a high level of apoptosis, concentrations were decreased to 1/5 of IC_50_ values for each single agent) and 2 μM and 0.05 μM (IB115). (**B**) Effects on cell cycle distribution. Quantification of cell cycle analysis (shown in Figure S4B) using propidium iodide incorporation and flow cytometry after 48 h of treatment using the same drug concentrations on the same cell lines as in (**A**). (**C**,**D**). Effects on long-term survival. Clonogenic assays in the WDLPS (93T449) and DDLPS cell lines (IB111, IB115) and in the control soft tissue LMS cell line (IB136), using the indicated JNJ42756493 and RG7388 concentrations. (**C**) Representative pictures of the clonogenic assay. (**D**) Quantification of the number of colonies obtained in the same experimental conditions as in (**C**).

We further analyzed the effect of the JNJ42756493 + RG7388 combination on apoptosis using an annexin V assay. Consistent with the isobologram experiments, we demonstrated a strong synergistic effect of the drug combination on apoptosis induction in all LPS cell lines (Figure 5A and Figure S4A). Whereas each drug alone induced apoptosis in 17% to 35% and 30% to 51% of the cells for JNJ42756493 and RG7388 as single drugs respectively, drug combination induced apoptosis in both WDLPS and DDLPS cells from 62% to massive apoptosis (89%) in the DDLPS IB115 cells.

We also investigated the effects of JNJ42756493 and RG7388 on cell cycle distribution. As shown in Figure 5B and Figure S4B, treatment with JNJ42756493 or/and RG7388 for 48 h led to cell cycle arrest in the G1 phase and a decrease in the proportion of cells in S phase in both WDLPS and DDLPS cells.

To conclude our in vitro studies, we examined the effects of JNJ42756493 and RG7388 on long-term survival using a clonogenic assay. Interestingly, JNJ42756493, when used at the IC_50_ values concentrations, completely inhibited colony formation in all three LPS cell lines. Our aim was to demonstrate that, in combination with RG7388, JNJ42756493 at very low concentrations could significantly inhibit colony formation in WDLPS and DDLPS cells. We chose to use low drug concentrations which produced only marginal effects when used as single agents: RG7388 from 5 to 70 nM and JNJ42756493 from 1 to 20 nM (Figure 5C,D). Indeed, when combined with RG7388 (5–70 nM), JNJ42756493 used at concentrations 100 times (for 93T449 and IB115), and even 1000 times (for IB111) lower than its IC_50_ values, was capable of inhibiting colony formation by 50%.

Importantly, when the LMS IB136 cells were treated with the JNJ42756493 and RG7388 combination using even higher concentrations than those used for the LPS cells, no synergy was observed (Figure 5C,D) indicating that the synergistic effect between JNJ42756493 and RG7388 was specific to the WDLPS and DDLPS histological subtypes.

### 2.5. Dual Targeting of FGFR and MDM2 In Vivo

**Figure 6:**
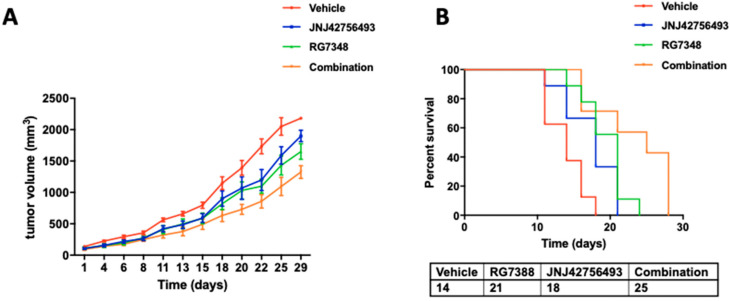
In vivo evaluation of the combination of JNJ42756493 and RG7388. (**A**) Tumor growth in mice injected with DDLPS IB115 cells and treated with either vehicle, RG7388, JNJ42756493, or a combination of the two drugs (combination). (**B**) Kaplan–Meier survival curves for the four cohorts. A log-rank (Mantel–Cox) test was used to calculate *p*-values comparing the survival rates.

To further validate the results of our in vitro study, we performed xenograft experiments testing the effects of the JNJ42756493 + RG7388 combination on in vivo tumor growth. IB115 cells were subcutaneously injected to Ragγ2C-/- mice to generate xenograft tumours. The mice were randomized into four groups including control (vehicle), JNJ42756493 (JNJ42756493 alone 40 mg/kg), RG7388 (RG7388 alone 12.5 mg/kg), and a combination of both drugs (JNJ42756493 + RG7388). We observed that the JNJ42756493 + RG7388 combination significantly reduced tumor growth rate (Figure 6A) without inducing toxicity (mice body weights were stable in all the four groups). Moreover, survival was also affected with a median of survival of 25 days for the combination treatment group, 14 days for the control group (*p* = 0.0016), 18 days for the JNJ42756493 group (*p* = 0.0288) and 21 days for the RG7388 group (*p* = 0.0443) (Figure 6B). 

## 3. Discussion

Our study is the first to provide an exhaustive analysis of the expression of the four FGFRs and their prognostic value in a very large series of nearly 700 WDLPS and DDLPS cases. Our series includes a majority of DDLPS cases in accordance with the need of efficient targeted therapies for patients with metastatic DDLPS. We have analyzed the expression of the four FGFRs at the protein level using IHC in 418 WDLPS and DDLPS cases. We have shown that a significant percentage of WDLPS and DDLPS overexpress FGFR1 and/or FGFR4 and that this expression was correlated with poor prognosis (Figure 1). Importantly, the results obtained at the protein level were validated at the mRNA level from the analysis of an independent public data set (Figure S1). Our findings therefore suggest that FGFR1 and FGFR4 expression may serve as predictive biomarkers allowing the stratification of patients for inclusion in FGFR inhibitor clinical trials. Several publications have discussed the important issue of *FGFR* amplification value versus mRNA and protein expression as biomarkers to predict efficacy of FGFR inhibitors [38]. *FGFR* amplification appears to be a rare event in WDLPS and DDLPS. Asano et al. used target sequencing with an Oncopanel including the four FGFRs to analyze a series of 19 WDLPS and 37 DDLPS cases [39]. They reported one DDLPS case harboring a mutation in *FGFR1* and 2 DDLPS cases with *FGFR3* amplification. Importantly, the authors did not detect any FGFR3 protein expression by IHC in the cases harboring *FGFR3* amplification [39]. In line with these results, arrayCGH analysis performed routinely as part of the diagnostic activity of our Laboratory of tumor genetics on 67 WDLPS and DDLPS samples detected only one DDLPS case showing *FGFR* amplification (*FGFR1*).

FGFR overexpression thus does not appear to result from *FGFR* amplification and might be the result of posttranscriptional or posttranslational regulation mechanisms. Note that FGFR overexpression without *FGFR* gene amplification has been described in other tumor types [38] and [40].

The role of tyrosine kinase genes in LPS growth has been investigated using siRNA and small-molecule inhibitor screening [41]. The authors have identified PTK2 and KIT as novel potential therapeutic kinase targets in DDLPS. They also demonstrated that the multi-targeted tyrosine kinase inhibitor ponatinib could be an effective drug candidate for LPS management; interestingly, ponatinib includes FGFRs among its targets. 

Regarding FGFRs, their role in tumorigenesis has been described in many different types of tumors including sarcomas [42,43,44,45]. In LPS, available reports on this topic are scarce and have mainly focused on the potential role of FRS2 in DDLPS tumorigenesis [17,23] on the basis of the nearly systematic amplification of the *FRS2* gene in these tumors [16,46,47]. However, a very recent study did not support the potential of *FRS2* amplification as a biomarker in DDLPS since DDLPS cell lines expressing the same levels of *FRS2* did not display the same sensitivity to the FGFR inhibitor LY2874455 [23]. Our data confirm the very frequent amplification of *FRS2* (96%) in both WDLPS and DDLPS primary tumors (CGH array analysis on 67 WDLPS and DDLPS cases). In our panel of LPS cell lines (one WDLPS and two DDLPS), we have detected mRNA overexpression, but it was not correlated with the protein levels (Figure 2A) in accordance with previous reports [16,17], suggesting post transcriptional regulation of *FRS2* expression in these cells. Importantly, we have shown that *FRS2* mRNA expression is not associated with prognosis (Figure S1G) in line with a previous report [47].

We detected predominant expression of FGFR1 and FGFR4 in our panel of WDLPS and DDLPS cell lines (as reported by Hanes et al. in three other DDLPS cell lines [23]), thereby reflecting FGFR expression pattern in primary tumors. In vitro experiments demonstrated a functional role of FGFR signaling in WDLPS and DDLPS cells. FRS2 phosphorylation dramatically increased upon addition of a FGFR ligand (FGF1) indicating that the FGFR pathway is functional in WDLPS and DDLPS cells (Figure 2B). We tested the activity of JNJ42756493, a potent and selective pan-FGFR inhibitor [25] currently approved for the management of bladder cancer [48]. Perera et al. have established the biochemical and functional selectivity of JNJ42756493 for FGFR1–4 with limited off-target activity against other highly related tyrosine kinases such as VEGFR2 [25]. Importantly, clinical data obtained so far regarding JNJ42756493 toxicity are consistent with the absence of significant VEGFR2 inhibitory activity of this inhibitor [49]. 

In all three cell lines, exposure to JNJ42756493 induced a decrease in viability, a cell cycle arrest in G1, and apoptosis. We detected high levels of ERK phosphorylation in unstimulated cells (Figure 2B) even in serum-deprived cells. JNJ42756493 treatment had a strong inhibitory effect on the ERK1/2 pathway (Figure 2C,D), indicating that the FGFR pathway plays a major role in ERK activation in WDLPS and DDLPS cells. The PI3K/AKT pathway was less affected (Figure 2C,D), confirming previous reports using other FGFR inhibitors (LY2874455 [23] and NVP-BGJ398 in DDLPS cells [17,24]). Importantly, the impact of FGFR signaling on survival appeared much more pronounced than the impact on proliferation. Indeed, JNJ42756493 concentrations required to observe a 50% reduction in the colony formation were in the nM range (10 nM, 200 nM, 36 nM for respectively IB111, IB115 and 93T449 cells) vs. values in the μM range for MTT proliferation experiments. Strikingly, the relevance of our pre-clinical data is supported by the data we provide on a patient with DDLPS refractory to multiple lines of treatment and whose tumor was stabilized for 12 weeks on JNJ42756493 (Figure 1G). Interestingly, the tumor was shown to overexpress FGFR1 and FGFR4 supporting our hypothesis. This patient displayed secondary resistance after three months of treatment. This suggests that a combination of therapies is necessary to overcome feedback loops and resistance; our study provides data on two combinations. The first one associating JNJ42756493 to the PI3K/mTOR antagonist BEZ235 unexpectedly proved to be antagonistic in DDLPS cells (Figure S3C). We have previously shown that combining RG7388 with targeted non-genotoxic drugs such as the CDK4 inhibitor palbociclib [35] or the MAPK kinase inhibitor GSK1120212 may hold therapeutic potential [37]. The second combination we tested, associating JNJ42756493 with RG7388, was found to exert a highly synergistic effect on all the tumorigenicity parameters we analyzed (Figure 5) in all three WDLPS and DDLPS cell lines. We showed a dramatic effect of the JNJ42756493 + RG7388 combination on apoptosis induction ranging from 65% to 89% (Figure 5A). Marked effects on long-term survival were observed for low, therefore more clinically relevant, concentrations of JNJ42756493 and RG7388 (Figure 5C,D).

## 4. Materials and Methods

### 4.1. Patients Population

Two retrospective series of clinical tumor samples (694 samples) from patients with WDLPS/DDLPS were studied for FGFR expression. The first series, analyzed at the protein level, included 418 WDLPS/DDLPS cases from 358 patients enrolled in the FILIPO (NCT03303885, https://clinicaltrials.gov/ct2/show/NCT03303885) multicentric study that involved three French sites (Centre Hospitalier Universitaire of Nice, Nice; Institut Bergonié, Bordeaux, and Centre Antoine Lacassagne, Nice), including 106 patients with WDLPS and 252 patients with DDLPS. All cases were diagnosed by pathologist experts in soft tissue and confirmed by detection of *MDM2* amplification, according to the WHO Classification of Soft Tissue and Bone Tumors [1,2]. Clinical, pathological, and follow-up data were available for all patients. All patients gave their written non-opposition consent. The second series, analyzed at the mRNA level, was used as an independent validation set. It included WDLPS/DDLPS cases from 276 patients and 11 normal fat samples. It corresponds to a sample subset included in a pooled series of 1432 primary soft tissue sarcomas that we had previously gathered from 16 public data sets [33]. All samples had been profiled using DNA microarrays or RNA Sequencing. Inclusion criteria of the 276 samples were WDLPS/DDLPS pathological subtype and availability of *FGFR1* and *FGFR4* expression data. Before analysis, data were normalized as previously described [33].

### 4.2. Analysis of FGFRs Protein Expression in Clinical Samples

All 418 cases of the first series were analyzed by IHC for expression of FGFRs, using the following monoclonal antibodies: FGFR1 (clone D8E4, 1/50, #9740, Cell Signaling Technology Danvers, MA, USA), FGFR2 (ab58201, 1/100, Abcam, Cambridge, MA, USA), FGFR3 (clone B-9, sc-13121, 1/100, Santa Cruz Biotechnology, Dallas, TX, USA), and FGFR4 (clone A-10, sc-136988, 1/50, Santa Cruz Biotechnology), using the Dako Autostainer (Agilent Technologies, Santa Clara, CA, USA), according to the manufacturer’s recommendations. Validation of the antibodies used for FGFR IHC analysis was performed using tissues exhibiting a molecular alteration for FGFR when available: *FGFR1* amplification in a DDLPS case (Figure S5A); *FGFR3* fusion in a glioblastoma case (Figure S5C). For FGFR2 and FGFR4, normal gastric mucosa was used (Figure S5B,D). For 282 cases from Institut Bergonié, included in the Réseau de Référence en Pathologie des Sarcomes (RRePS) database [50], a tissue micro-array containing three representative spots for each case was constructed from formalin-fixed paraffin-embedded tissue (FFPE). For each one of the remaining cases, a representative whole-tissue section was analyzed from FFPE tissue.

Cytoplasmic or membranous staining was scored by two pathologists (BDM-JFM) according to the staining intensity (0: absence of staining; 1: weak staining; 2: moderate staining; and 3: strong staining) and the percentage of tumor cells harboring this staining intensity. The score of immunostaining for each FGFR ranged from 0 to 300, as a result of multiplying the percentage of positive tumor cells by the staining intensity. Cut-off values for continuous percentages and staining intensities were determined on predicting patient OS and DFS. Cut-off values of 50 and 5 were selected for, respectively, FGFR1 and FGFR4 protein expression because they were the most discriminative in predicting patient OS and DFS using the biostatistical analysis described in the statistical analysis section. Overexpression was therefore defined as tumors with a score superior or equal to 50 for FGFR1 (score ≥ 50) and a score superior or equal to 5 for FGFR4 (score ≥ 5).

### 4.3. Cell Lines and Culture Conditions

All cell lines were established in our laboratories (Solid Tumor Genetics Laboratory of Nice University Hospital, Nice, France and Institut Bergonié, Bordeaux, France) from a primary retroperitoneal WDLPS (93T449), a primary periscapular DDLPS (IB111), a primary paratesticular DDLPS (IB115), and a soft-tissue leiomyosarcoma (LMS) (IB136) [51]. All cell lines were genomically characterized by array-CGH and tested for mycoplasma using the VenorGeM Mycoplasma Detection Kit (Minerva Biolabs, Berlin, Germany). The cells were cultured in RPMI-1640 medium containing GlutaMAX (Life Technologies, Waltham, MA, USA) supplemented with 10% fetal bovine serum (Life Technologies, Waltham, MA, USA) and 1% penicillin-streptomycin (Sigma-Aldrich, St. Louis, MO, USA) and grown at 37 °C, with 5% CO_2_.

### 4.4. Real-Time Quantitative Polymerase Chain Reaction (qRT-PCR) Analysis

RNA was isolated from cells using Trizol (Invitrogen). The quantification and quality of the isolated RNA were evaluated using respectively a Nanodrop 2000 (ThermoFisher, Waltham, MA, USA) and the 2100 Bioanalyzer (RNA 6000 Nano kit) (Agilent Technologies, Santa Clara, CA, USA). RNA samples were treated by DNA-free (Applied Biosystems, Foster City, CA, USA). For each sample, one microgram of total RNA was reverse-transcribed into cDNA using the High capacity cDNA Reverse transcription kit (Applied Biosystems, Foster City, CA, USA). The following TaqMan gene expression assays (Applied Biosystems) were used MDM2: Hs01066938_m1; FGFR1: Hs00241111_m1; FGFR2: Hs01552918_m1; FGFR3: Hs00179829_m1; FGFR4: Hs00242558_m1; FRS2: Hs00183614_m1; RPLP0: Hs99999902_m1. RPLP0 was used as endogenous control for normalization. Short-term culture of dermolipectomy cells was used as the reference sample. The comparative threshold cycle (Ct) method was used to achieve relative quantification of gene expression. Each qRT-PCR experiment was performed three times in duplicate with the ABI PRISM 7500 detection System using FAM dyes (Applied Biosystems) according to the manufacturer’s recommendations.

### 4.5. Drugs

The pan-FGFR inhibitor JNJ42756493 (erdafitinib) was supplied by Janssen Pharmaceuticals (Beerse, Belgium), the MDM2-TP53 interaction inhibitor RG7388 (idasanutlin) was supplied by Roche (Roche Pharma Research and Early Development, Basel, Switzerland), and the dual PI3K/mTOR inhibitor BEZ235 (dactolisib) was purchased from Selleckchem (Houston, TX, USA). 

### 4.6. Drug Synergy Assays

The drug synergy assays were performed as previously described, using the isobologram and combination index (CI) methods, derived from the Chou and Talalay principle [35,36]. The combination effect of the two treatments was defined as follows: CI < 1, synergistic effect, CI = 1, additive effect, and CI > 1, antagonistic effect.

### 4.7. Cell Viability, Cell Apoptosis, and Cell Cycle Assays

All cell viability, cell apoptosis, and cell cycle assays were performed as previously described [35,52].

### 4.8. Clonogenic Cell Survival Assay

Cells were seeded at very low density (330 cells per 10 cm-plate). The colonies were allowed to grow for 21 days in growth complete medium with specific drugs. After fixation in formaldehyde and staining with crystal violet (Sigma-Aldrich), the plates were scanned and the number of cell colonies was counted using FIJI (FIJI is just ImageJ) software. 

### 4.9. Western Blotting

Cells were treated for 45 min with either JNJ42756493 or control-treated with the corresponding concentration of JNJ427756493 diluent DMSO and for the last 15 min with or without 10 ng/mL of recombinant FGF1 (PeproTech, #100–17A, Rocky Hill, NJ, USA). Treated and control cells were then washed with PBS and were extracted using a 1.5 X Laemmli lysis buffer. Whole cell proteins (30 μg) were electrophoresed on 8–10% SDS-PAGE gels and transferred to PVDF membranes (Immobilon, Millipore, Burlington, MA, USA). Membranes were blocked with 5% BSA7030 (Sigma-Aldrich), for phosphorylated FRS2 or low-fat milk, then incubated overnight at 4°C with the following antibodies: anti-phospho-ERK (Thr202/Tyr204, #4370), anti-ERK (137F5, #4695), anti-phospho-AKT (Ser473, #4060), anti-AKT (11E7, #4685), anti-FGFR1 (D8E4, #9740), anti-FGFR4 (D3B12, #8562), anti-FGFR2 (D4L2V, #23328), anti-FGFR3 (C51F2, #4574), and anti-phosphoFRS2α (Tyr436, #3861) from Cell Signaling Technologies, anti-FRS2 (ab183492, Abcam), anti-MDM2 (Ab1-IF2, OP46, Calbiochem, Darmstadt, Germany), anti-P53 (DO-1, sc-126, Santa Cruz Biotechnology), anti-P21WAF1 (Ab-1, EA-10, Millipore), anti-βtubulin (66240–1-Ig, ProteinTech, Rosemont, IL, USA), and anti-GAPDH (60004–1-Ig, ProteinTech).

Quantification of the WB was performed using ImageJ software. Images are representative of the results obtained in three separate experiments.

### 4.10. Animal Studies

Four- to five-week-old female Ragγ2C-/- mice were used. Induction of tumor xenografts was performed by subcutaneous injection of 0.2 mL cell suspensions containing 5 × 10^6^ live IB115 cells into the right flank of the mice. This study followed the French and European Union guidelines for animal experimentation (RD 1201/05, RD 53/2013 and 86/609/CEE, respectively). Mice were randomized into control and treatment groups (*n* = 9 for vehicle, *n* = 10 for JNJ42756493, RG7388 groups and for combination group) two weeks after the tumor became measurable (15 days after injection: day 1 of treatment). Mice were randomized in 4 groups: vehicle (RG7388 diluent), RG7388 alone (12.5 mg/kg oral gavage five times per week), JNJ42756493 alone (40 mg/kg oral gavage five times per week), and both drugs (RG7388 at 12.5 mg/kg and JNJ42756493 at 40 mg/kg, five times per week). RG7388 and JNJ42756493 were administered using RG7388 diluent (Klucel2%, Tween 80 0.1%, Methylparaben 0.09%, propylparaben 0.01%) and 2-hydroxypropyl beta-cyclodextrin 10% as the vehicle respectively. The tumors were measured every 2–3 days with a caliper, and diameters were recorded. Tumor volumes were calculated using the formula: a^2^b/2, where a and b are the 2 largest diameters. The mice were sacrificed by cervical dislocation when tumors reached 2000 mm^3^. Survival curves were established based on the time between the beginning of treatment and sacrifice of mice. All experimental manipulations with mice were performed under sterile conditions in a laminar flow hood. 

### 4.11. Statistical Analysis

Data are represented as the mean +/- standard deviation (SD) from at least three experiments. Differences between groups were analyzed by one-way analysis of variance (ANOVA) or Student *t*-tests. Significant differences are indicated as * *p* < 0.05 ** *p* < 0.01 *** *p* < 0.001. Analysis of progression-free survival in animal studies was made using a log-rank test (Mantel–Cox test). All statistical analyses were performed with GraphPad Prism version 7.0 statistical software.

For the biostatistical analysis of expression data, qualitative data were described by using absolute and relative frequencies, and quantitative data were described by using mean, standard deviation, or median and range as appropriate. The continuous values were compared between groups using the Student *t*-test. Overall survival was defined as the time from primitive sarcoma diagnosis to death. Disease-free survival was defined as the time from sarcoma diagnosis to either local or metastatic recurrence. Patients showing no event (death or progression) or lost to follow-up were censored at the date of their last contact. Statistical comparisons were performed using a Cox regression model for survival data. All significant variables in univariate analysis were included in the multivariate models. By definition, grading (according to the Fédération Nationale des Centres de Lutte Contre le Cancer (FNCLCC) criteria) is correlated with subtypes, so only subtypes are included in the multivariate analysis. Statistical analyses were two-sided and were performed using R-3.5.1 for Windows. Cut-off values for FGFR1 and FGFR4 expression versus survival data were evaluated by using R function “cutp” for survival data model from the R package “survMisc” (http://www.rdocumentation.org/packages/survMisc/versions/0.5.5/topics/cutp). The “cutp” function was bootstrapped (*n* = 1000 replications) and the optimal cut-off corresponded to the median value calculated from these 1000 replications. Cut-off values of 50 and 5 were selected for respectively FGFR1 and FGFR4 protein expression because they were the most discriminative in predicting patient OS and DFS. When multiple samples were analyzed for one patient, only the first event was considered for statistical analyses (primitive event vs. recurrence or metastasis).

## 5. Conclusions

In conclusion, effective well-tolerated treatments for DDLPS in the advanced/metastatic setting are lacking. FGFR inhibitors are reported to induce manageable toxicities in patients. Our findings provide a rationale to use FGFR1 and FGFR4 protein expression as biomarkers to select patients for clinical trials investigating FGFR inhibitors. Furthermore, we have demonstrated the efficacy of the JNJ42756493 + RG7388 combination both in vitro and in vivo; we hope that our data can be transferred to the clinical setting.

## Figures and Tables

**Table 1:** Distribution of FGFR1 and FGFR4 immunohistochemical expression in our series of 418 cases of WDLPS and DDLPS.

FGFR1 and FGFR4 Expression	All LPS (*n* = 418)	WDLPS (*n* = 127)	DDLPS (*n* = 291)
FGFR1 expression			
<50	304 (73%)	113 (89%)	191 (66%)
≥50 *	114 (27%)	14 (11%)	100 (34%)
FGFR4 expression			
<5 ≥5 *	307 (73%) 111 (27%)	109 (86%) 18 (14%)	198 (68%) 93 (32%)
FGFR1 and FGFR4 status			
FGFR1 < 50 and FGFR4 < 5	237 (57%)	100 (79%)	137 (47%)
FGFR1 < 50 and FGFR4 ≥ 5	67 (16%)	13 (10%)	54 (19%)
FGFR1 ≥ 50 and FGFR4 < 5	70 (17%)	9 (7%)	61 (21%)
FGFR1 ≥ 50 and FGFR4 ≥ 5 ^§^	44 (11%)	5 (4%)	39 (13%)

* overexpression; ^§^ co-overexpression FGFR1 and FGFR4.

## Supplementary Materials

The following are available online at https://www.mdpi.com/2072-6694/12/10/3058/s1 Figure S1. *FGFR1* and *FGFR4* mRNA expression in WDLPS/DDLPS. Figure S2. Effect of JNJ42756493 on 93T449 (WDLPS), IB111 (DDLPS) and IB115 (DDLPS) cell viability. Figure S3. Effect of BEZ235 on activation of the p42/p44 MAP kinase and AKT pathways and effect of the JNJ42756493 and BEZ235 combination on in vitro tumorigenicity. Figure S4. Apoptosis and cell cycle profiles in 93T449, IB111 and IB115 cells treated with the JNJ42756493 and RG7388 combination. Figure S5. Validation of the anti-FGFR antibodies used for the IHC study. Table S1. Patients’ clinico-pathological characteristics from the cohort analyzed at the protein level (*n* = 358). Table S2. Survival cox analyses. Table S3: Clinicopathological characteristics of 276 samples analyzed at the mRNA level. Table S4. Determination of IC_50_ and Combination Index values. Supplementary Material. Full blots and quantifications.
