# Supplementary material for: Novel Therapeutic Insights in Dedifferentiated Liposarcoma: A Role for FGFR and MDM2 Dual Targeting
Source: Cancers (Basel). 2020 Oct 20;12(10):3058. doi: 10.3390/cancers12103058 (PMC7589658; doi:10.3390/cancers12103058)
Supplement: Supplementary file 1 [file cancers-12-03058-s001.zip › supplementary material revised.pptx]

## Slide 1
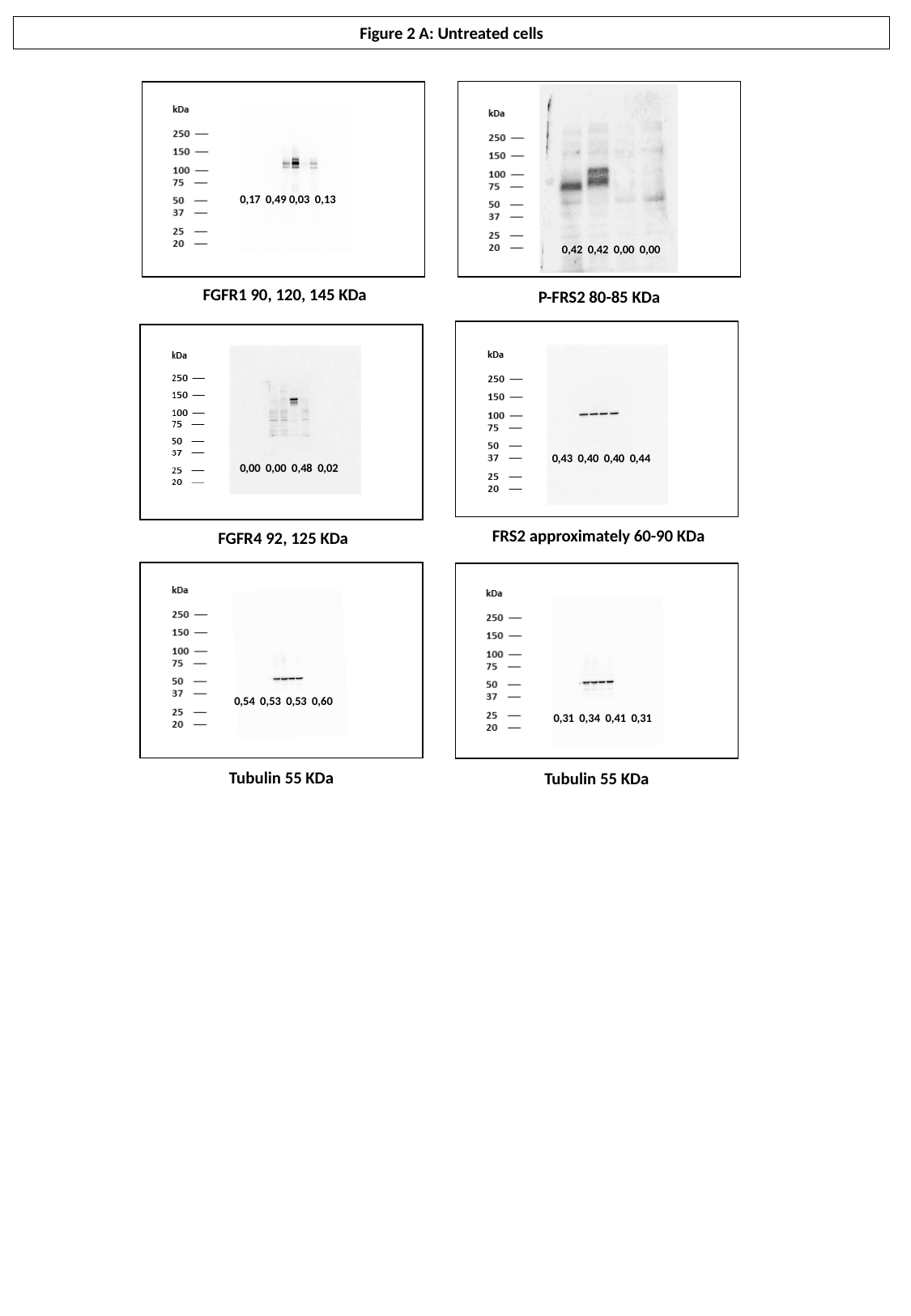

Figure 2 A: Untreated cells
0,17 0,49 0,03 0,13
0,42 0,42 0,00 0,00
kDa
250 
150 
100 
75 
50 
37 
25 
20 
FGFR1 90, 120, 145 KDa
P-FRS2 80-85 KDa
0,43 0,40 0,40 0,44
0,00 0,00 0,48 0,02
FRS2 approximately 60-90 KDa
FGFR4 92, 125 KDa
0,54 0,53 0,53 0,60
0,31 0,34 0,41 0,31
Tubulin 55 KDa
Tubulin 55 KDa

## Slide 2
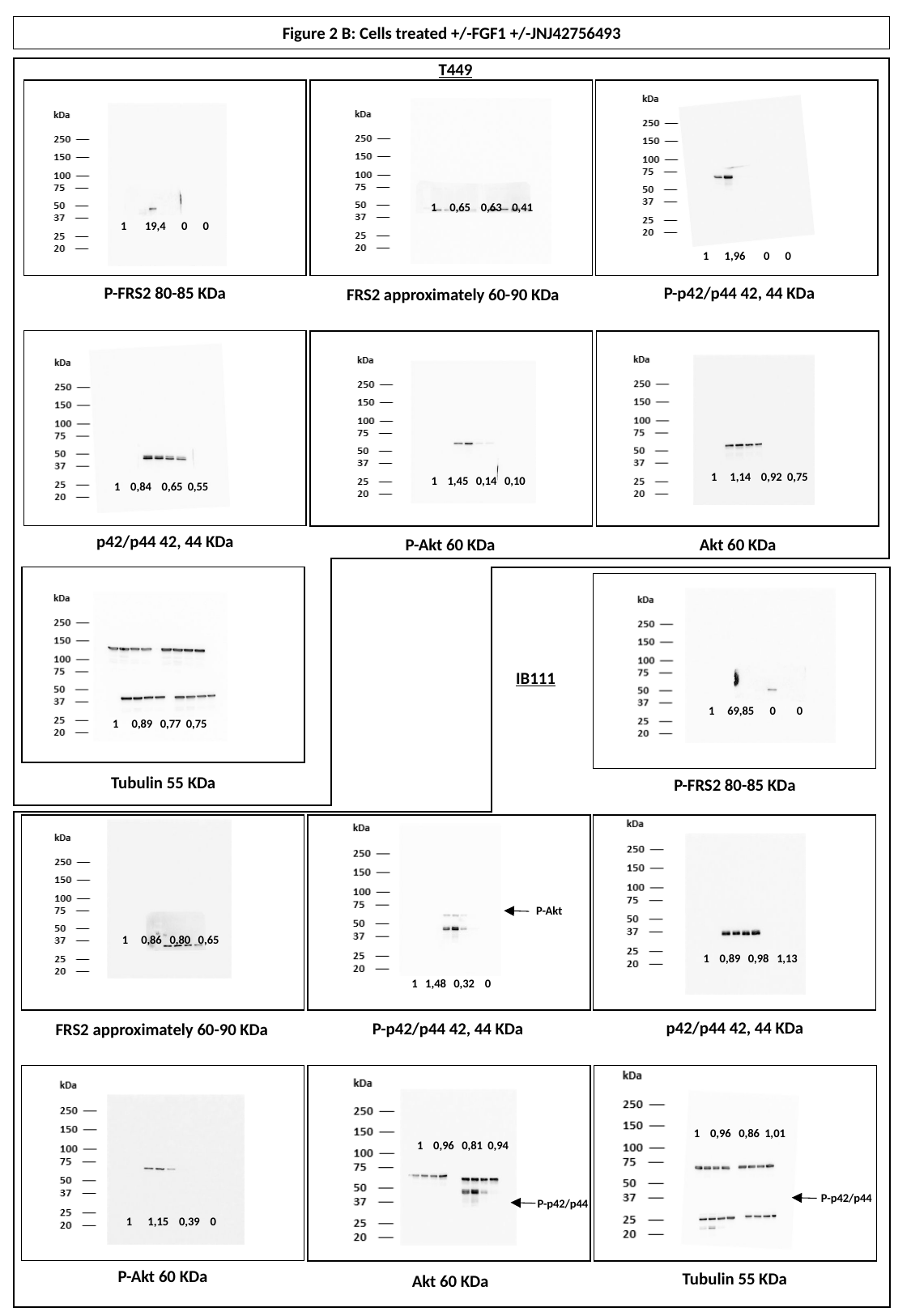

Figure 2 B: Cells treated +/-FGF1 +/-JNJ42756493
T449
 1 0,65 0,63 0,41
1 19,4 0 0
 1 1,96 0 0
P-FRS2 80-85 KDa
P-p42/p44 42, 44 KDa
FRS2 approximately 60-90 KDa
 1 1,14 0,92 0,75
 1 1,45 0,14 0,10
 1 0,84 0,65 0,55
p42/p44 42, 44 KDa
Akt 60 KDa
P-Akt 60 KDa
IB111
 1 69,85 0 0
 1 0,89 0,77 0,75
Tubulin 55 KDa
P-FRS2 80-85 KDa
P-Akt
 1 0,86 0,80 0,65
 1 0,89 0,98 1,13
 1 1,48 0,32 0
p42/p44 42, 44 KDa
P-p42/p44 42, 44 KDa
FRS2 approximately 60-90 KDa
0,43 0,40 0,40 0,44
 1 0,96 0,86 1,01
 1 0,96 0,81 0,94
P-p42/p44
P-p42/p44
 1 1,15 0,39 0
P-Akt 60 KDa
Tubulin 55 KDa
Akt 60 KDa

## Slide 3
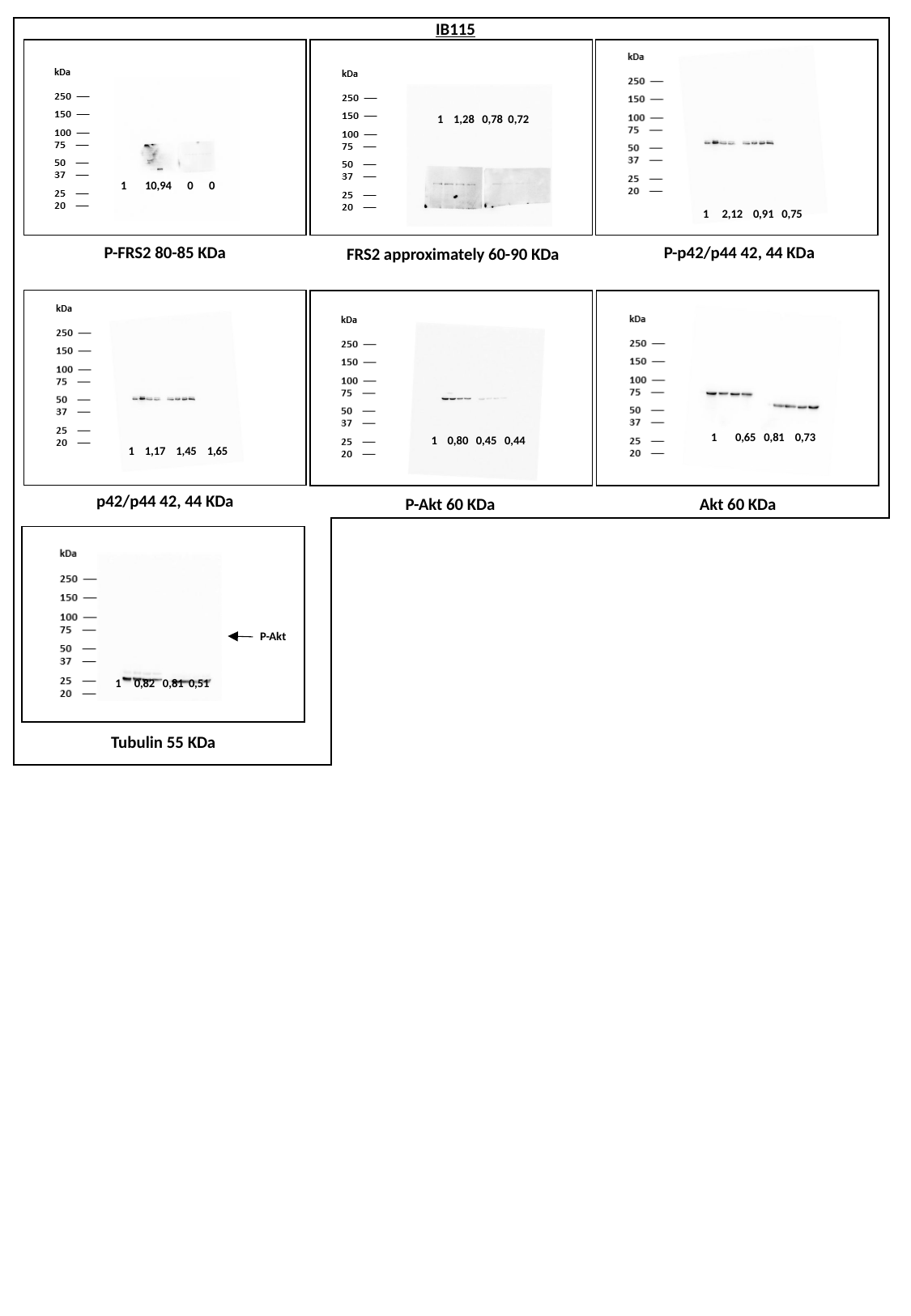

IB115
 1 1,28 0,78 0,72
1 10,94 0 0
 1 2,12 0,91 0,75
P-FRS2 80-85 KDa
P-p42/p44 42, 44 KDa
FRS2 approximately 60-90 KDa
 1 0,65 0,81 0,73
 1 0,80 0,45 0,44
 1 1,17 1,45 1,65
p42/p44 42, 44 KDa
Akt 60 KDa
P-Akt 60 KDa
P-Akt
 1 0,82 0,81 0,51
Tubulin 55 KDa

## Slide 4
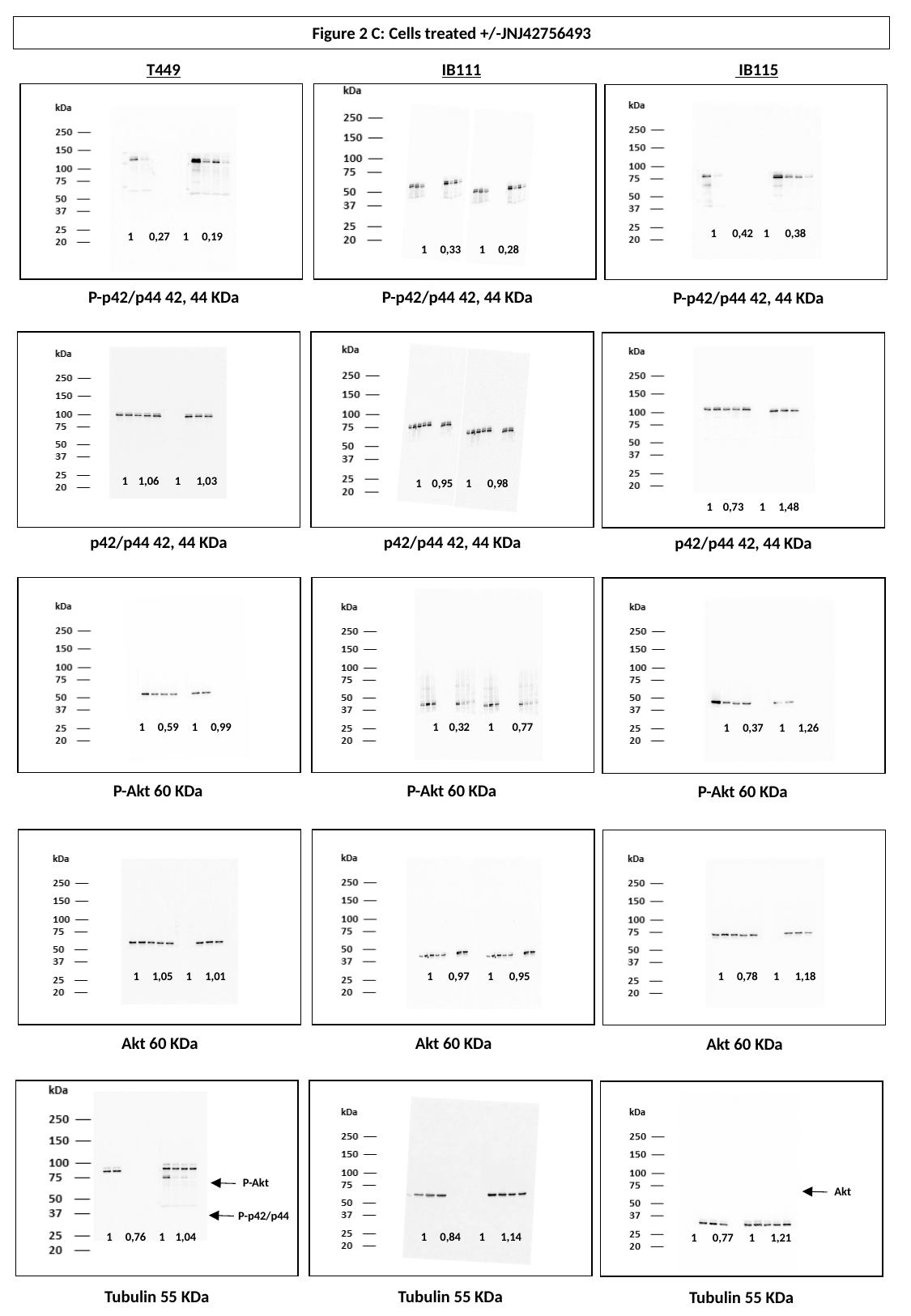

Figure 2 C: Cells treated +/-JNJ42756493
 T449 IB111 IB115
 1 0,42 1 0,38
 1 0,27 1 0,19
 1 0,33 1 0,28
P-p42/p44 42, 44 KDa
P-p42/p44 42, 44 KDa
P-p42/p44 42, 44 KDa
 1 1,06 1 1,03
 1 0,95 1 0,98
 1 0,73 1 1,48
p42/p44 42, 44 KDa
p42/p44 42, 44 KDa
p42/p44 42, 44 KDa
 1 0,32 1 0,77
 1 0,59 1 0,99
 1 0,37 1 1,26
P-Akt 60 KDa
P-Akt 60 KDa
P-Akt 60 KDa
 1 0,97 1 0,95
 1 1,05 1 1,01
 1 0,78 1 1,18
Akt 60 KDa
Akt 60 KDa
Akt 60 KDa
P-Akt
Akt
P-p42/p44
 1 0,84 1 1,14
 1 0,76 1 1,04
 1 0,77 1 1,21
Tubulin 55 KDa
Tubulin 55 KDa
Tubulin 55 KDa

## Slide 5
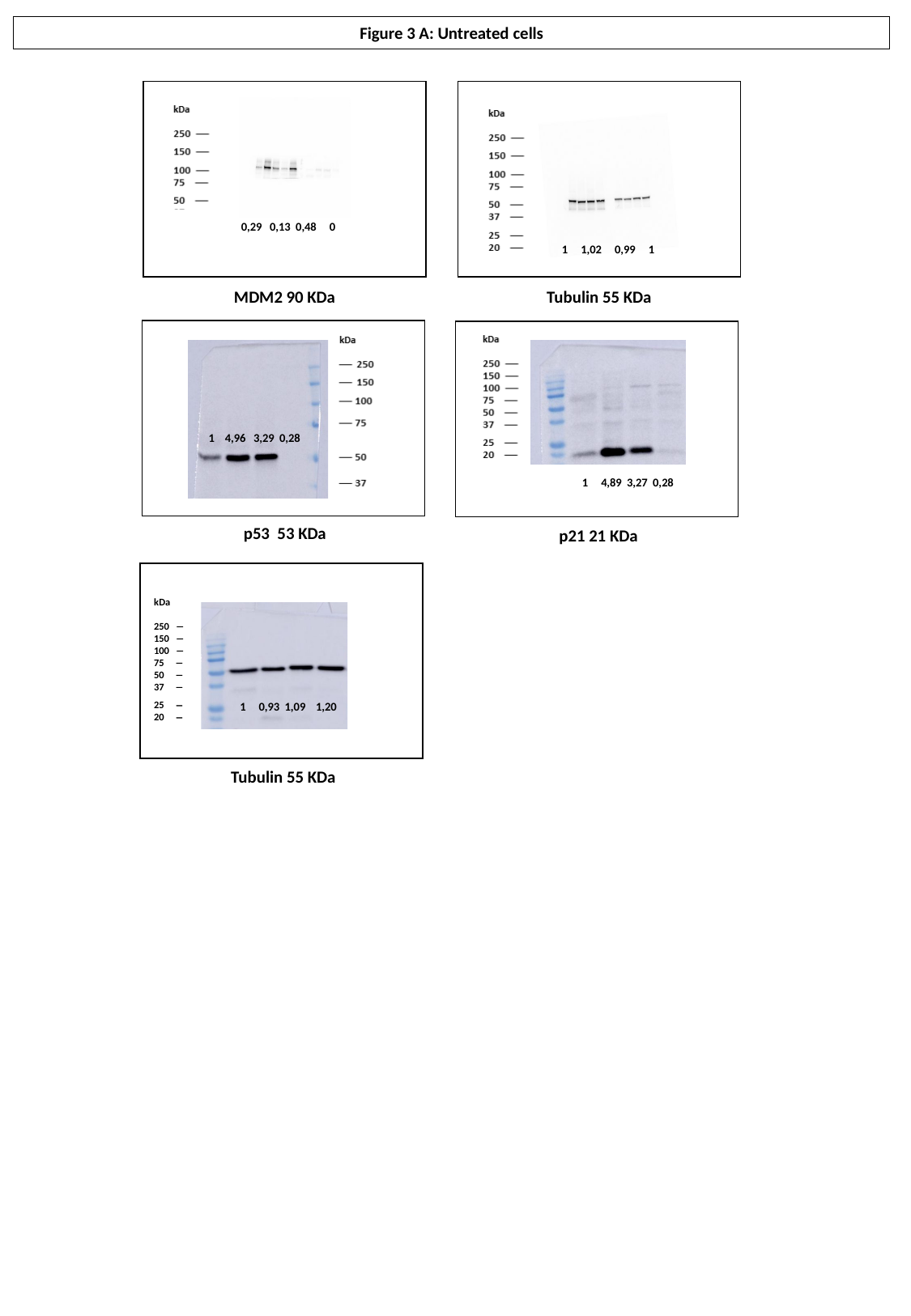

Figure 3 A: Untreated cells
0,29 0,13 0,48 0
1 1,02 0,99 1
MDM2 90 KDa
Tubulin 55 KDa
 1 4,96 3,29 0,28
 1 4,89 3,27 0,28
p53 53 KDa
p21 21 KDa
kDa
250 
150 
100 
75 
50 
37 
25 
20 
1 0,93 1,09 1,20
Tubulin 55 KDa

## Slide 6
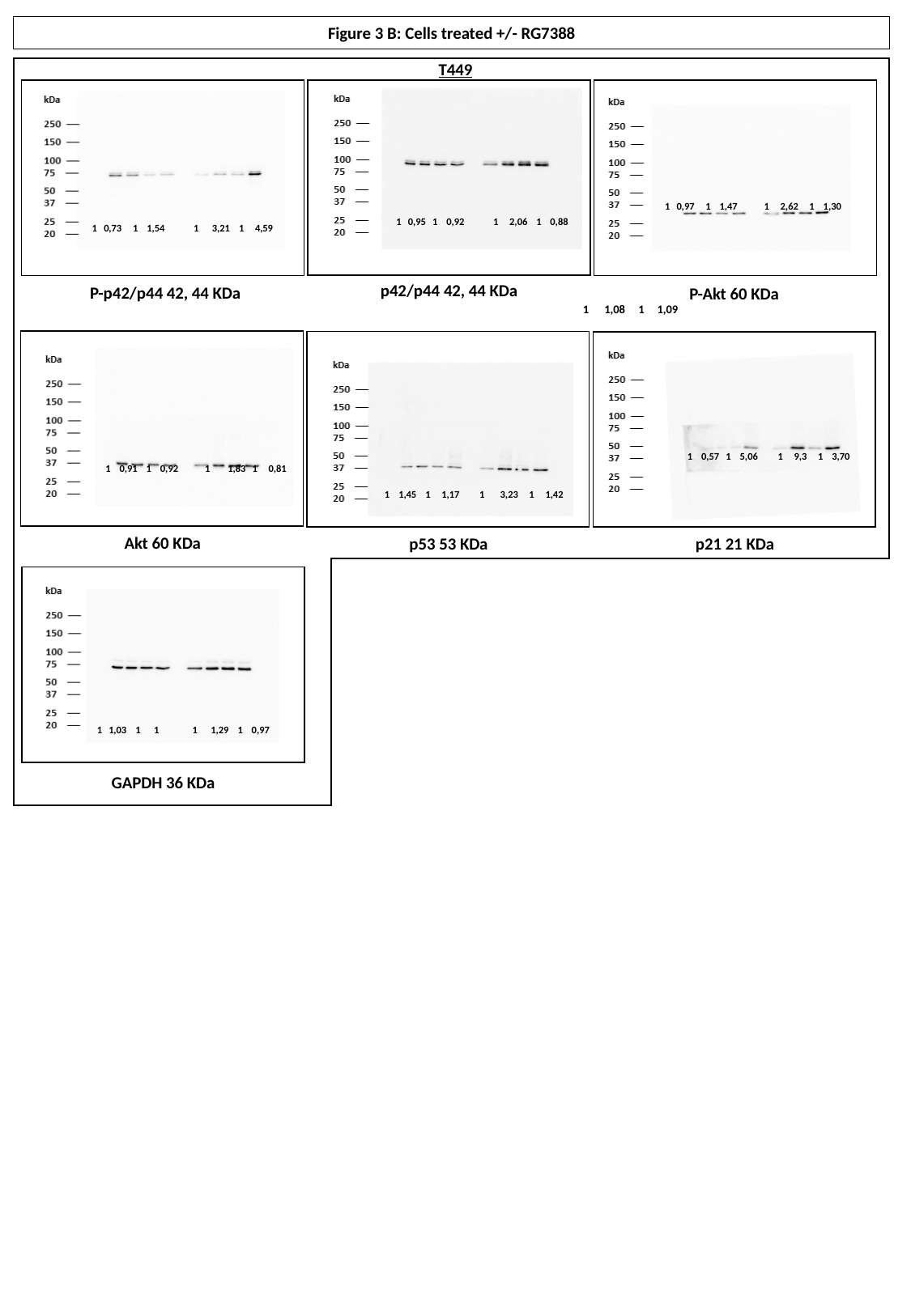

Figure 3 B: Cells treated +/- RG7388
T449
 1 0,97 1 1,47 1 2,62 1 1,30
 1 0,95 1 0,92 1 2,06 1 0,88
 1 0,73 1 1,54 1 3,21 1 4,59
p42/p44 42, 44 KDa
P-p42/p44 42, 44 KDa
P-Akt 60 KDa
 1 1,08 1 1,09
 1 0,57 1 5,06 1 9,3 1 3,70
 1 0,91 1 0,92 1 1,83 1 0,81
 1 1,45 1 1,17 1 3,23 1 1,42
Akt 60 KDa
p53 53 KDa
p21 21 KDa
 1 1,03 1 1 1 1,29 1 0,97
GAPDH 36 KDa

## Slide 7
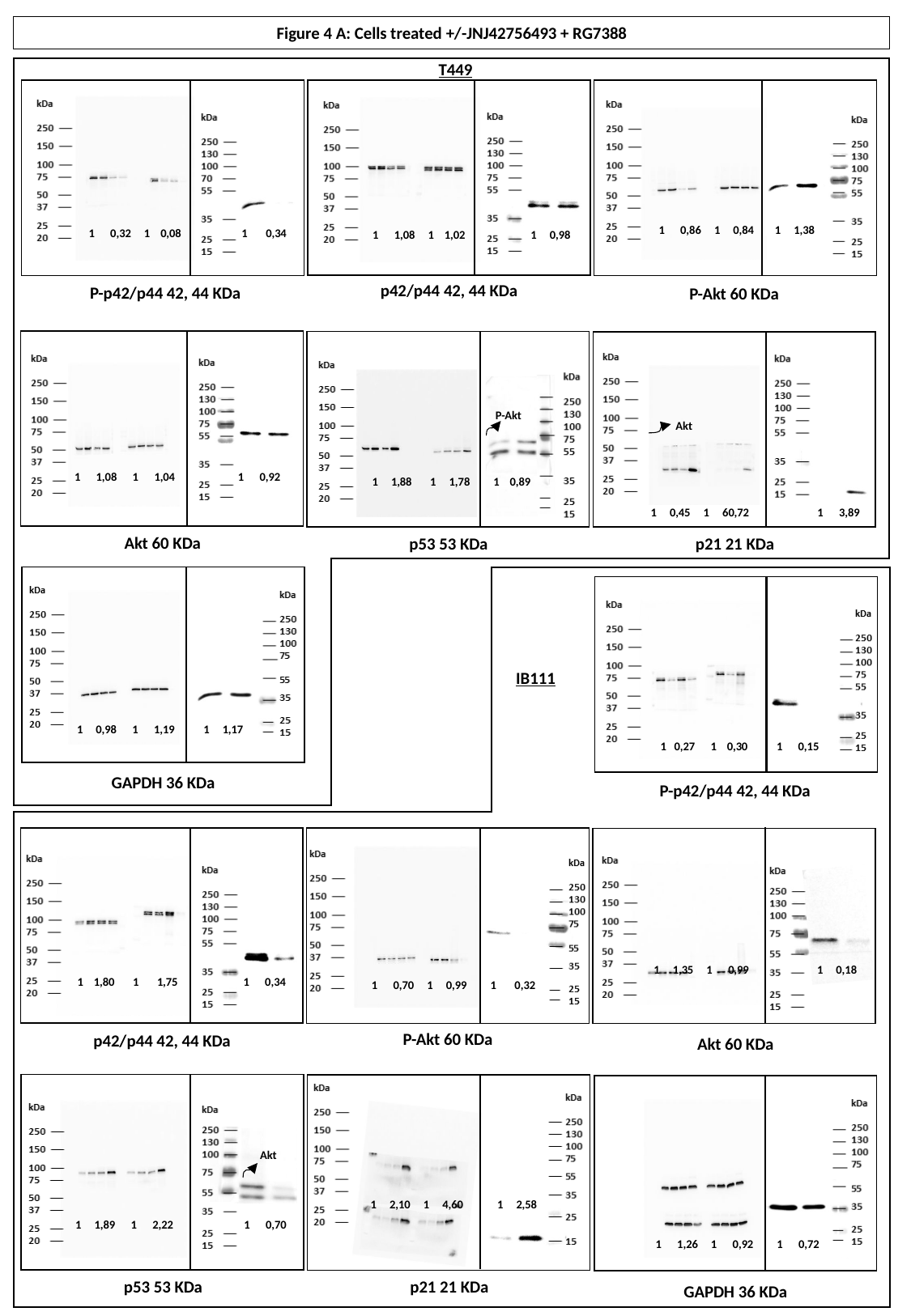

Figure 4 A: Cells treated +/-JNJ42756493 + RG7388
T449
 1 0,86 1 0,84 1 1,38
 1 0,32 1 0,08 1 0,34
 1 1,08 1 1,02 1 0,98
p42/p44 42, 44 KDa
P-p42/p44 42, 44 KDa
P-Akt 60 KDa
P-Akt
Akt
 1 1,08 1 1,04 1 0,92
 1 1,88 1 1,78 1 0,89
 1 0,45 1 60,72 1 3,89
Akt 60 KDa
p53 53 KDa
p21 21 KDa
IB111
 1 0,98 1 1,19 1 1,17
 1 0,27 1 0,30 1 0,15
GAPDH 36 KDa
P-p42/p44 42, 44 KDa
 1 1,35 1 0,99 1 0,18
 1 1,80 1 1,75 1 0,34
 1 0,70 1 0,99 1 0,32
P-Akt 60 KDa
p42/p44 42, 44 KDa
Akt 60 KDa
 Akt
 1 2,10 1 4,60 1 2,58
 1 1,89 1 2,22 1 0,70
 1 1,26 1 0,92 1 0,72
p53 53 KDa
p21 21 KDa
GAPDH 36 KDa

## Slide 8
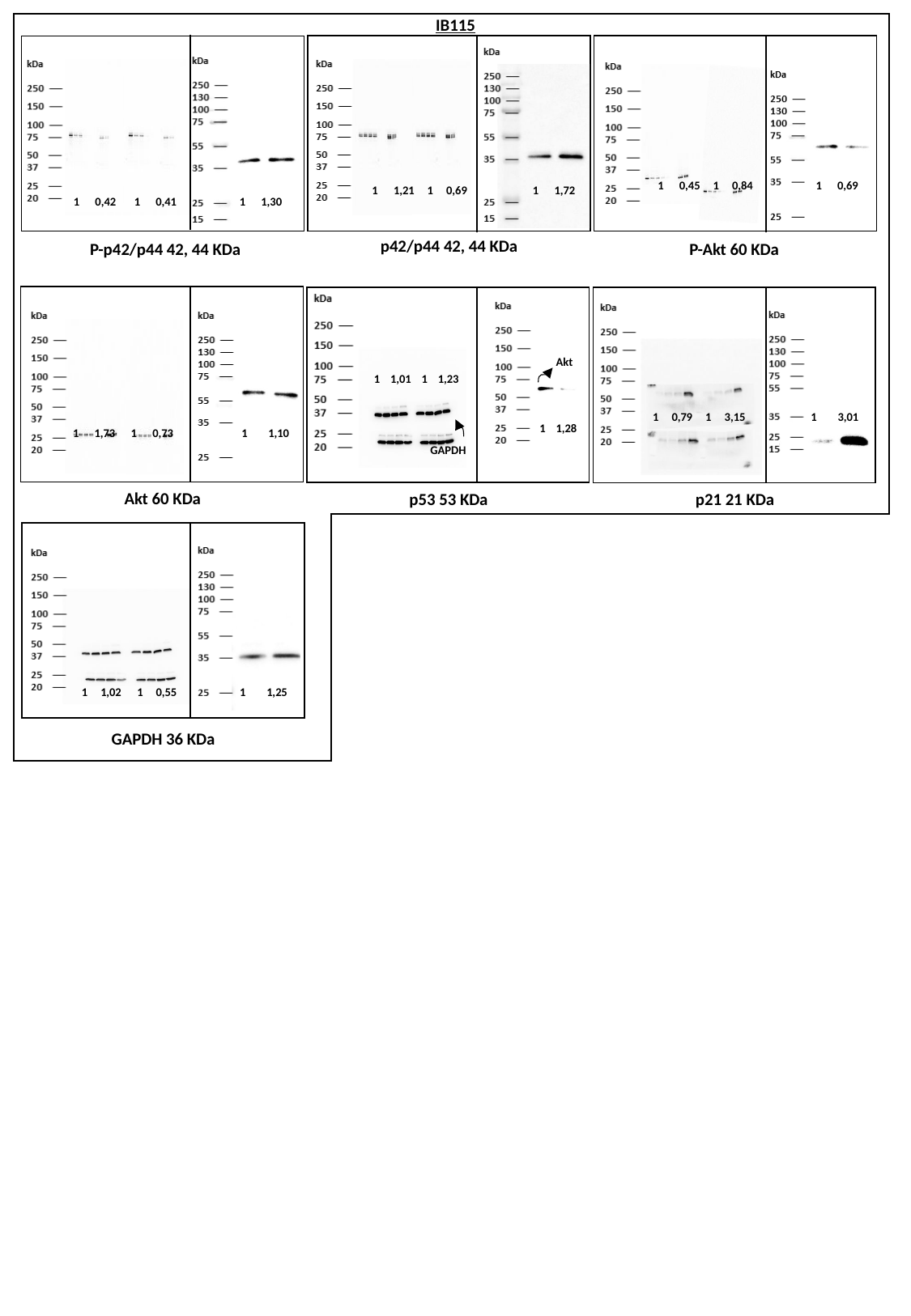

IB115
 1 0,45 1 0,84 1 0,69
 1 1,21 1 0,69 1 1,72
 1 0,42 1 0,41 1 1,30
p42/p44 42, 44 KDa
P-p42/p44 42, 44 KDa
P-Akt 60 KDa
 Akt
 1 1,01 1 1,23
 1 0,79 1 3,15 1 3,01
 1 1,28
 1 1,73 1 0,73 1 1,10
GAPDH
Akt 60 KDa
p53 53 KDa
p21 21 KDa
 1 1,02 1 0,55 1 1,25
GAPDH 36 KDa

## Slide 9
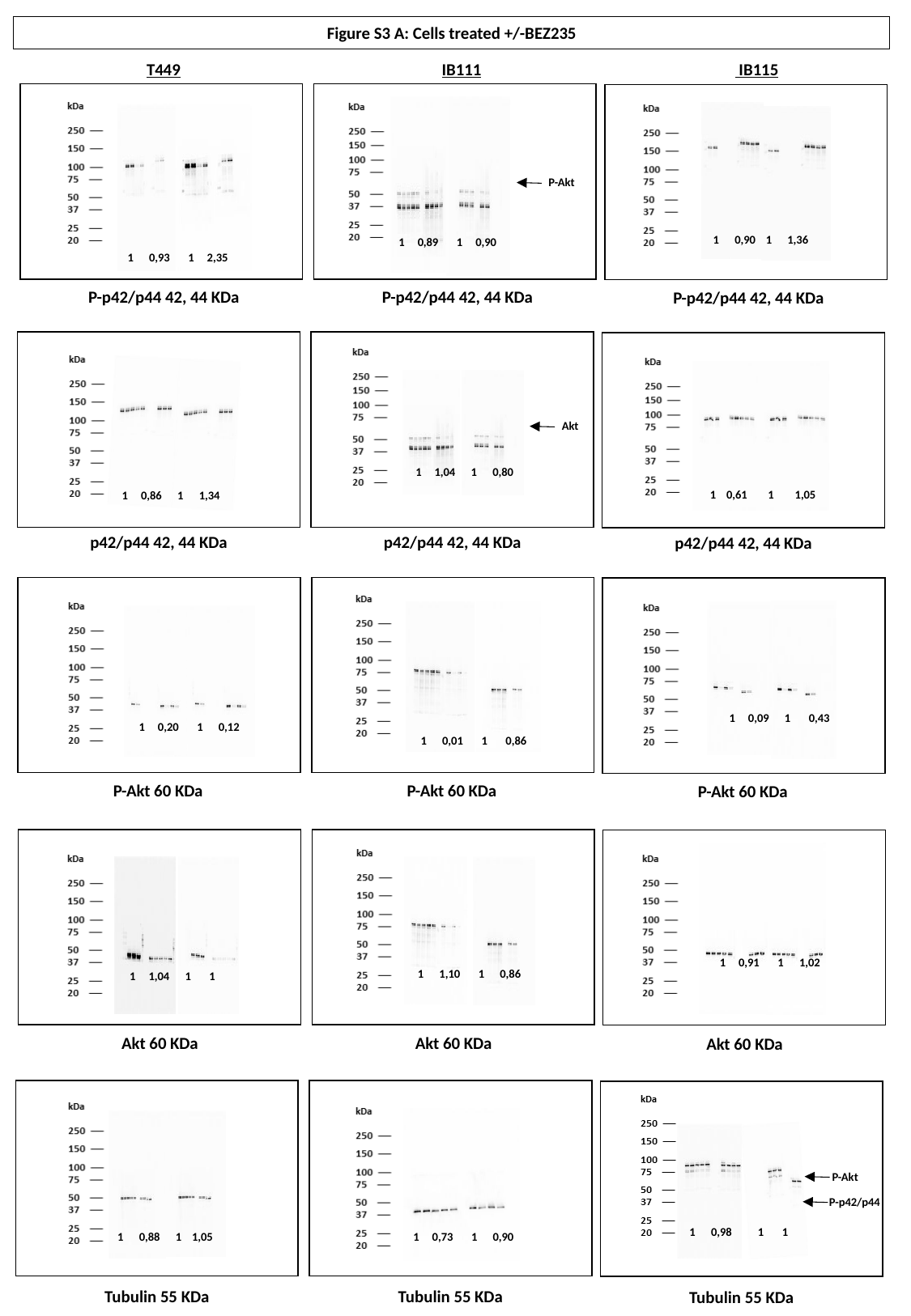

Figure S3 A: Cells treated +/-BEZ235
 T449 IB111 IB115
P-Akt
 1 0,90 1 1,36
 1 0,89 1 0,90
 1 0,93 1 2,35
P-p42/p44 42, 44 KDa
P-p42/p44 42, 44 KDa
P-p42/p44 42, 44 KDa
Akt
 1 1,04 1 0,80
 1 0,61 1 1,05
 1 0,86 1 1,34
p42/p44 42, 44 KDa
p42/p44 42, 44 KDa
p42/p44 42, 44 KDa
 1 0,09 1 0,43
 1 0,20 1 0,12
 1 0,01 1 0,86
P-Akt 60 KDa
P-Akt 60 KDa
P-Akt 60 KDa
 1 0,91 1 1,02
 1 1,10 1 0,86
 1 1,04 1 1
Akt 60 KDa
Akt 60 KDa
Akt 60 KDa
P-Akt
P-p42/p44
 1 0,98 1 1
 1 0,73 1 0,90
 1 0,88 1 1,05
Tubulin 55 KDa
Tubulin 55 KDa
Tubulin 55 KDa
