# Supplementary material for: Novel Therapeutic Insights in Dedifferentiated Liposarcoma: A Role for FGFR and MDM2 Dual Targeting
Source: Cancers (Basel). 2020 Oct 20;12(10):3058. doi: 10.3390/cancers12103058 (PMC7589658; doi:10.3390/cancers12103058)
Supplement: Supplementary file 1 [file cancers-12-03058-s001.zip › SupplementaryTableS1.docx]

| Characteristics | N (%) |
| --- | --- |
| Median age, years (n=358) | 64.2 (22-88)* |
| Sex (n=358)  Male  Female | 218 (61%)  140 (39%) |
| Samples (n=358)  Primitive tumor  Recurrence  Metastasis | 283 (79%)  69 (19%)  6 (2%) |
| Liposarcoma subtypes (n=358)  Well-differentiated (WDLPS)  Dedifferentiated (DDLPS) | 106 (30%)  252 (70%) |
| Location (n=352)  Limbs  Retroperitoneum  Head and neck  Other | 76 (22%)  221 (62%)  6 (2%)  49 (14%) |
| Grading (FNCLCC§) (n=297)  1  2  3 | 76 (26%)  152 (51%)  69 (23%) |
| Metastatic liposarcoma at diagnosis (n=358)  No  Yes | 350 (98%)  8 (2%) |
| Surgery: complete resection (R0) (n=283)  No  Yes | 233 (82%)  50 (18%) |
| Radiotherapy (n=350)  No  Yes | 199 (57%)  151(43%) |
| Chemotherapy (n=346)  No  Yes | 234 (68%)  112 (32%) |
| Targeted therapy (n=350)  No  Yes | 337 (96%)  13 (4%) |

**Supplementary Table S1. Patients’ clinico-pathological characteristics from the cohort analysed at the protein level (n=358).**

*, median (range)

§, Sarcoma grading according to the « Fédération Nationale des Centres de Lutte Contre le Cancer »
