# Supplementary material for: Novel Therapeutic Insights in Dedifferentiated Liposarcoma: A Role for FGFR and MDM2 Dual Targeting
Source: Cancers (Basel). 2020 Oct 20;12(10):3058. doi: 10.3390/cancers12103058 (PMC7589658; doi:10.3390/cancers12103058)
Supplement: Supplementary file 1 [file cancers-12-03058-s001.zip › SupplementaryTableS2.docx]

|  | HR† | CI95% | p-Cox |
| --- | --- | --- | --- |
| **Cox univariate analysis and disease-free survival (DFS)** | | | |
| Surgery: complete resection (R0)  Yes  No | 1  1.3 | REF.  [0.8-2.0] | -  0.317  -  <0.0001****  -  0.141  -  <0.001***  <0.0001****  -  0.0349 *  -  <0.0001**** |
| Liposarcoma subtypes  Well-differentiated (WDLPS)  Dedifferentiated (DDLPS) | 1  2.5 | REF.  [1.8-3.6] |  |
| Location  Limbs  Other | 1  1.3 | REF.  [0.9-2.0] |  |
| Grading (FNCLCC§)  1  2  3 | 1  2.3  3.5 | REF.  [1.5-3.5]  [2.1-5.6] |  |
| FGFR1 expression  <50  >=50 | 1  1.4 | REF.  [1.0-2.0] |  |
| FGFR4 expression  <5  >=5 | 1  2.2 | REF.  [1.6-3.1] |  |
| FGFR1+FGFR4 co-overexpression  FGFR1<50 + FGFR4<5  FGFR1>=50 + FGFR4>=5 | 1  2.9 | REF.  [1.8-4.9] | -  <0.0001**** |
| **Cox multivariate analysis and disease-free survival (DFS)‡** | | | |
| Liposarcoma subtypes  Well-differentiated (WDLPS)  Dedifferentiated (DDLPS) | 1  2.3 | REF.  [1.6-3.3] | -  <0.0001 |
| FGFR1 expression  <50  >=50 | 1  1.1 | REF.  [0.8-1.6] | -  0.48 |
| FGFR4 expression  <5  >=5 | 1  1.9 | REF.  [1.4-2.7] | -  0.00015 |
| FGFR1+FGFR4 co-overexpression  No  Yes | 1  2.1 | REF.  [1.3-3.4] | -  0.0025 |
| **Cox univariate analysis and overall survival (OS)‡** | | | |
| Surgery: complete resection (R0)  Yes  No | 1  2.7 | REF.  [1.2-5.7] | -  0.0132 * |
| Liposarcoma subtypes  Well-differentiated (WDLPS)  Dedifferentiated (DDLPS) | 1  2.8 | REF.  [1.9-4.3] | -  <0.0001**** |
| Location  Limbs  Other | 1  1.7 | REF.  [1.1-2.7] | -  0.024 * |
| Grading (FNCLCC§)  1  2  3 | 1  2.4  4 | REF.  [1.5-3.9]  [2.3-7] | -  <0.001***  <0.0001**** |
| FGFR1 expression  <50  >=50 | 1  2.1 | REF.  [1.5-3] | -  <0.0001**** |
| FGFR4 expression  <5  >=5 | 1  2.3 | REF.  [1.6-3.4] | -  <0.0001**** |
| FGFR1 + FGFR4 co-overexpression  FGFR1<50 and FGFR4<5  FGFR1>=50 and FGFR4>=5 | 1  4.7 | REF.  [2.6-8.4] | -  <0.0001**** |
| **Cox multivariate analysis and overall survival (OS) ‡** | | | |
| Surgery: complete resection (R0)  Yes  No | 1  2.50 | REF.  [1.2-5.4] | -  0.0206 |
| Liposarcoma subtypes  Well-differentiated (WDLPS)  Dedifferentiated (DDLPS) | 1  2.69 | REF.  [1.5-4.7] | -  0.00062 |
| Location  Limbs  Other | 1  1.56 | REF.  [0.91-2.66] | -  0.099 |
| FGFR1 expression  <50  >=50 | 1  1.9 | REF.  [1.2-3.0] | -  0.0080 |
| FGFR4 expression  <5  >=5 | 1  2.0 | REF.  [1.2-3.1] | -  0.00618 |
| FGFR1+FGFR4 co-overexpression  No  Yes | 1  4.0 | REF.  [2.1-7.8] | -  <0.0001 |

**Supplementary Table S2. Survival cox analyses.** †, Hazard ratio; §, Sarcoma grading according to the « Fédération Nationale des Centres de Lutte Contre le Cancer »; **‡,** FNCLCC grading not included in the model because of correlation with subtypes.
