# Supplementary material for: Novel Therapeutic Insights in Dedifferentiated Liposarcoma: A Role for FGFR and MDM2 Dual Targeting
Source: Cancers (Basel). 2020 Oct 20;12(10):3058. doi: 10.3390/cancers12103058 (PMC7589658; doi:10.3390/cancers12103058)
Supplement: Supplementary file 1 [file cancers-12-03058-s001.zip › SupplementaryTableS3.docx]

| Characteristics | N = 276 |
| --- | --- |
| Age (years) | 63 (35-87)* |
| Sex  Male  Female | 49 (67%)  24 (33%) |
| Liposarcoma subtypes  Well-differentiated (WDLPS)  Dedifferentiated (DDLPS) | 58 (21%)  218 (79%) |
| Location  Limbs  Internal trunk  Head and neck  Superficial trunk | 5 (5%)  88 (87%)  1 (1%)  7 (7%) |
| Grading (FNCLCC§)  1-2  3 | 1(2%)  58 (98%) |
| Pathological tumor size, median (cm) | 18.4 (1.2-39.5) |
| Follow-up median (months) | 30 (1-222) |
| DFS events, number of patients | 70 (35%) |
| 5-year DFS % [95%CI] | 57% [49-66] |

**Supplementary Table S3: Clinicopathological characteristics of 276 samples analysed at the mRNA level.**

*, median (range)

§, Sarcoma grading according to the « Fédération Nationale des Centres de Lutte Contre le Cancer »
