# Supplementary material for: Novel Therapeutic Insights in Dedifferentiated Liposarcoma: A Role for FGFR and MDM2 Dual Targeting
Source: Cancers (Basel). 2020 Oct 20;12(10):3058. doi: 10.3390/cancers12103058 (PMC7589658; doi:10.3390/cancers12103058)
Supplement: Supplementary file 1 [file cancers-12-03058-s001.zip › SupplementaryTableS4.docx]

|  | **93T449**  **WDLPS** | **IB111**  **DDLPS** | **IB115**  **DDLPS** |
| --- | --- | --- | --- |
| **JNJ42756493**  IC_50_ value* | 0.36 μM | 1 μM | 2 μM |
| **RG7388**  IC_50_ value* | 0.04 μM | 0.2 μM | 0.05 μM |
| **JNJ42756493 + RG7388**  Combination Index§ | 0.18  **Synergy** | 0.40  **Synergy** | 0.26  **Synergy** |
| **BEZ235**  IC_50_ value* | 0.05 μM | 0.01 μM | 0.06 μM |
| **JNJ42756493 + BEZ235**  Combination Index§ | 0.75  **Synergy** | 2.3  Antagonism | 1.35  Antagonism |

**Supplementary Table S4. Determination of IC_50_ values and Combination Indexes**

*, determined using MTT assay; §, calculated according to the Chou and Talalay method
